# Heterologous *codA* Gene Expression Leads to Mitigation of Salt Stress Effects and Modulates Developmental Processes

**DOI:** 10.3390/ijms241813998

**Published:** 2023-09-12

**Authors:** Galina N. Raldugina, Lilia R. Bogoutdinova, Olga V. Shelepova, Vera V. Kondrateva, Ekaterina V. Platonova, Tatiana L. Nechaeva, Varvara V. Kazantseva, Pyotr V. Lapshin, Helen I. Rostovtseva, Tatiana S. Aniskina, Pyotr N. Kharchenko, Natalia V. Zagoskina, Alexander A. Gulevich, Ekaterina N. Baranova

**Affiliations:** 1 K.A. Timiryazev Institute of Plant Physiology, Russian Academy of Sciences, 127276 Moscow, Russia; nechavtatyana.07@yandex.ru (T.L.N.); vkazantceva90@gmail.com (V.V.K.); p.lapshin@mail.ru (P.V.L.); ni-fir-titi@mail.ru (H.I.R.); zagoskina@mail.ru (N.V.Z.); 2 All Russia Research Institute of Agricultural Biotechnology, Russian Academy of Sciences, 127550 Moscow, Russiaiab@iab.ac.ru (P.N.K.); a_gulevich@mail.ru (A.A.G.); 3 N.V. Tsitsin Main Botanical Garden of Russian Academy of Sciences, Botanicheskaya 4, 127276 Moscow, Russialab-physiol@mail.ru (V.V.K.); tatianiskina@gmail.com (T.S.A.); 4 Limited Liability Company “NPP Biosfera”, Vernadskogo 96, 119571 Moscow, Russia

**Keywords:** *Nicotiána tabácum*, glycine betaine, choline oxidase, transgenic plants, salinity, metabolism, stress tolerance

## Abstract

Transgenic tobacco plants overexpressing the choline oxidase gene from *A. globiformis* showed an increase in resistance at the level of primary and secondary biosynthesis of metabolites, removing the damage characteristic of salinity and stabilizing the condition of plants. We used 200 mM NaCl, which inhibits the growth of tobacco plants at all stages of development. Leaves of transgenic and wild-type (WT) plants *Nicotiána tabácum* were used for biochemical, cytological and molecular biological analysis. However, for transgenic lines cultivated under normal conditions (without salinity), we noted juvenile characteristics, delay in flowering, and slowing down of development, including the photosynthetic apparatus. This caused changes in the amount of chlorophyll, a delay in the plastid grana development with the preservation of prolamellar bodies. It also caused changes in the amount of sugars and indirectly downstream processes. A significant change in the activity of antioxidant enzymes and a change in metabolism is probably compensated by the regulation of a number of genes, the expression level of which was also changed. Thus, the tolerance of transgenic tobacco plants to salinity, which manifested itself as a result of the constitutive expression of *codA*, demonstrates an advantage over WT plants, but in the absence of salinity, transgenic plants did not have such advantages due to juvenilization.

## 1. Introduction

One of the most effective and widespread mechanisms of protection against abiotic stresses is the increase and regulation of osmoprotectant synthesis. The most promising compound from these osmolytes, along with proline, is a quaternary amine produced from choline by a number of enzymes—glycine betaine (GB). This compound is found in a wide range of animals, bacteria and some drought and salt-tolerant angiosperms [1,2]. In natural accumulators such as spinach, corn, sugar beet, and barley, rapid GB accumulation occurs during exposure to salt, drought, and low-temperature stresses [3,4]. GB protects plants by acting as an osmolyte, maintaining the water balance between plant cells and the environment, and stabilizing macromolecules during cell dehydration and high salt concentrations, i.e., acting as a molecular chaperone [5,6]. To date, transgenic plants containing the choline oxidase gene involved in the one-step synthesis of GB have already been created [7,8,9,10,11,12,13,14]. Plants producing this osmolyte showed resistance not only to salts but also to various other stresses, such as hypothermia, freezing, high temperature and drought [2,15]. Physiological studies have shown that the level of accumulated GB correlates with the degree of salt tolerance [16]. At the same time, if the GB accumulation using the transit peptide was targeted at chloroplasts, then this led to a higher resistance of plants to stress than when the compound was located in the cytosol, because transgenic plants genetically engineered with the *codA* gene, whose product targets chloroplasts, accumulate glycine-betaine mainly in these organelles [12,17]. The accumulation of glycine betaine in the chloroplasts of transgenic plants effectively protects the photosynthetic apparatus under salt stress [17].

To study the effectiveness of protection against stress-induced damage, we created a construct with a semisynthetic choline oxidase gene that promotes a one-step converting choline to glycine betaine process, in contrast to the two-step synthesis system naturally present in plants. In this study, we report the study of tolerance to NaCl salt of previously obtained glycine betaine-synthesizing tobacco plants obtained by genetic transformation with a construct with the *codA* gene from *Arthrobacter globiformis* [18] (Figure S1). The enzyme provided with a signal sequence is delivered to the plastid and starts a process leading to an increase in osmotic pressure, which contributes to the protection of cellular components and, accordingly, plant protection [19].

The aim of this study was to assess in detail the morphological and physiological response of transgenic tobacco plants overexpressing the choline oxidase gene from *Arthrobacter globiformis* to salt stress, as well as to study the expression of some genes associated with adaptation to soil salinity. The main hypothesis was to evaluate the modulation of biochemical synthesis responsible for primary and secondary metabolism in the targeted protection of the plastid compartment by the synthesis of an osmotically active compound and to show that transgenic plants with an increased pool of glycine betaine would show greater tolerance to saline soil conditions. 

## 2. Results

### 2.1. Comparative Characteristics of codA Transgenic and WT Plants

**Figure 1:**
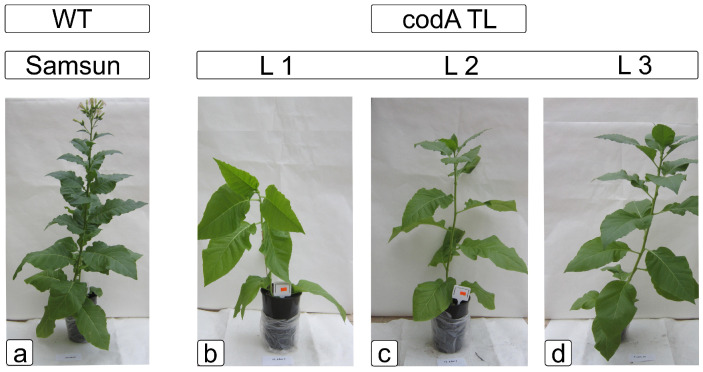
Phenotypic differences between wild-type plants (**a**) and transgenic *codA* lines (**b**–**d**) in soil culture.

Tobacco (*Nicotiana tabacum* L.) transgenic lines (L1, L2, L3) together with WT were used to test *codA* function for salt tolerance. Plants with heterologous *codA* expression did not grow as well under control conditions and had some obvious phenotypic changes within three weeks of planting. WTs were dark green, stood taller, and formed generative organs. The transgenic *codA* lines were lower, had no buds, and had a lettuce color (Figure 1).

We noted that the glycine betaine sample content in the previously selected transgenic lines did not differ significantly from each other. In the control plants, an insignificant amount of glycine betaine was noted, which is possibly due to the peculiarities of the sample preparation method (Figure S1).

The electrical conductivity of the solution indicates the total amount of electrolytes in the tissues of the studied plants. Control tobacco leaves had a higher electrical conductivity than leaves of transgenic tobacco plants. This means that Samsun tobacco plants had a higher total electrolyte content (1.3–1.4 times) compared to leaves of tobacco lines 1–3 (Figure S2a). With almost the same level of potassium and sodium ions and a slightly lower content of chloride ions (Figure S2b–d).

We were unable to establish significant differences in third leaf size and water content between transgenic and nontransgenic leaves (Figure S3).

Meanwhile, the width of the stomatal gap in transgenic plants was significantly higher (Figure S3c,d). The width of the stomatal opening of the stomata of the lower epidermis was lower (in lines 1 and 3) or comparable in line 2 (Figure S3d). To some extent, this corresponded to and possibly was caused by differences in the size of the stomata of the upper (Figure S3e) and lower (Figure S3f) epidermis, which in transgenic plants were also larger in the upper epidermis and smaller in the lower, respectively (stomata in Figure S3e,f).

To control stress induction, we examined the level of the “stress hormone” ABA in transgenic and WT plants without NaCl exposure, the data did not differ significantly (Figure S4).

Differences in color, expressed in a paler color of the transgenic plant leaves, we confirmed the expected significant decrease in the content of chlorophyll a and b, respectively, as well as their sum (Figure S4b,c). The indicators of the ratio of chlorophylls a/b were ambiguous, in transgenic line L2 they did not differ from WT, while in L1 and L3 they were higher (Figure S4d).

Malonic dialdehyde, being a toxic compound capable of interacting with free amino groups of proteins, was used for additional analysis of possible damage caused by *codA* overexpression. However, its level was significantly lower in transgenic lines (Figure S4e). At the same time, line 2 had even lower values, also retaining the previously noted differences.

To understand the effect of glycine betaine on the main osmotic parameter, which is highly dynamic, especially in response to stress and salinity, we analyzed the content of the amino acid proline. Interestingly, the proline level of the L2 transgenic line did not differ from the control, while the L1 and L3 lines had significantly higher values (Figure S4f).

A special role in maintaining the osmotic potential and the availability of chemical energy accumulated as a result of photosynthesis is played by primary metabolites obtained as a result of photosynthetic processes of carbon fixation, free sugars used in the plant body for accumulation in the form of starch, transportation, and synthesis of cell wall polysaccharides. The drop in sugar levels in the leaves of transgenic lines was significant compared to WT, and the lines also differed from each other (Figure S5a). Line L2 had the lowest value.

The amount of phenolic compounds in the transgenic lines was markedly reduced (Figure S5b). The values of flavonoids were somewhat lower; however, they were significantly lower only in line L3 (Figure S5c).

Phenolic acids are involved in the occurrence of induced plant resistance to adverse environmental conditions. In our work, we found a significant increase in chlorogenic, and caffeic acids in transgenic plants (Figure S5d,e). However, ferulic acid present in WT plants was not found in transgenic lines.

The plant’s antioxidant system is a powerful stabilizing link in protection against adverse effects. We found that the transgenic lines showed a significant imbalance, the activity of GPOD guaiacol peroxidase was reduced, with a significant increase in the activity of superoxide dismutases (Figure S5f,g).

### 2.2. Comparative Characteristics of codA Transgenic and WT Plants under Saline Conditions

To assess phenotypic changes, we assessed the degree of transition to generative development, expressed in the formation of an inflorescence, the transition to flowering, as well as the state of turgor and the severity of green color by plant phenotyping.

**Figure 2:**
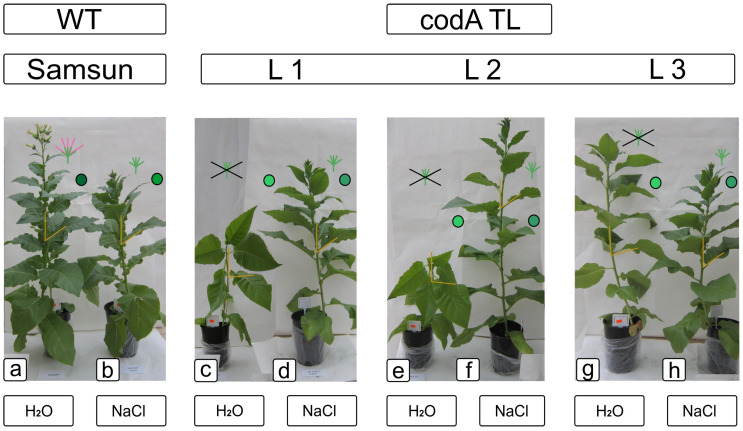
Phenotypic differences between wild-type plants (**a**,**b**) and transgenic *codA* lines (**c**–**h**) in soil culture without and after treatment with 200 mM NaCl. Legend: orange lines petiole angle; circle—the severity of the green color; scheme of transition to generative development. The cross means the absence of generative organs.

**Figure 3:**
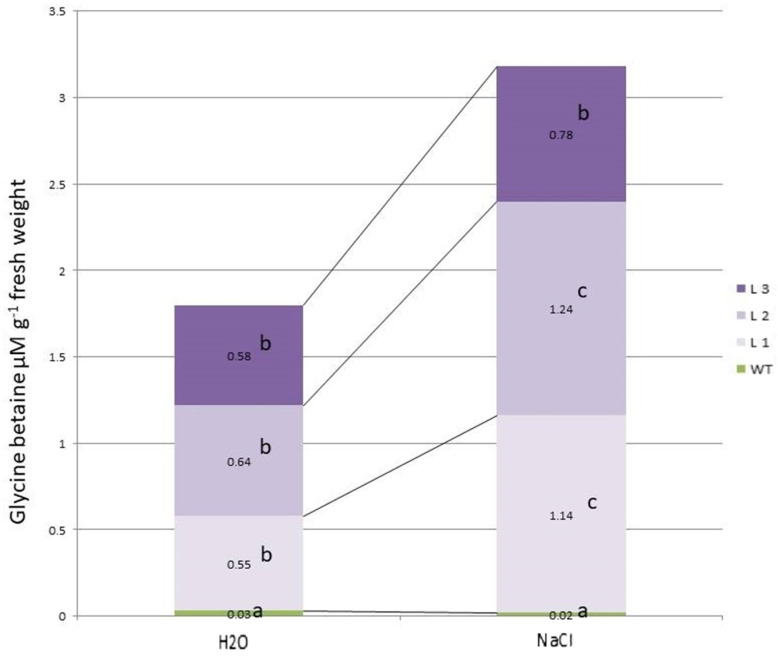
Glycine betaine content in *codA* transgenic plant lines. Control—Tc medium without adding NaCl; NaCl—Tc medium with the addition of 200 mM NaCl. Columns with the same letters are not significantly different (*p* ≤ 0.05, Duncan’s test).

Transgenic and WT plants showed different responses to salinity both at the phenotypic (Figure 2, Figure S6) and physiological and biochemical levels. The effect of stress led to an increase in the synthesis of glycine betaine only in transgenic lines L1 and L2, while in L3 the amount of glycine betaine did not increase, and in control WT plants, the pool of glycine betaine was insignificant and did not change with salinity (Figure 3). 

The quantification of visually noticeable differences between the lines of transgenic plants and the wild type under various cultivation conditions (when irrigated with water and with the NaCl addition) shows that data on color phenotyping, petiole emergence, and changes in the stage of ontogeny are associated with the influence of choline oxidase and are sensitive to the action of salinity (Figure S6).

Changes in plant growth and the angle of departure of leaf petioles from the stem are reflected in the histograms (Figure S7).

Other phenotyping data such as RGB were used to evaluate relationships in correlation analysis with other parameters (Figure S8).

It was found that the *NtAct9PBL* expression was significantly associated with plant height only in L3 in the control variant, the correlation was strong and direct (r = 0.98). Expression of *NtP5CS A*, *NtDHN*, and *ChSYNPBL* did not correlate with plant height. Strong expression of the *SODLC1* in L2 in the control variant (water) significantly inhibited growth (r = −0.9), while it had the opposite effect (r = 0.98) in L3 treated with NaCl. Expression of *NtAct9PBL* was manifested only in WT and L3 in the control variant (water) and was associated only with the Green component in RGB (Red-Green-Blue) phenotyping, in both cases the correlation was strong and positive (i.e., the more expression of this gene, the “greener” there will be plants). Expression of *NtP5CS A* had feedback associated with Red and Blue (RGB) in L1 (i.e., as expression increases, plants lose red and blue and appear greener). Only in the L1, *NtDHN* expression had a strong positive relationship with all color scores (r = 0.98 for red blue and green). *SODLC1* expression was strongly associated with Green in L3 under NaCl treatment (since r = −0.95, increasing the expression of this gene reduces the amount of green). *ChSYNPBL* expression was quite strongly associated with Blue in WT under NaCl treatment (r = 0.9). *CodA* expression was quite strongly associated with Blue in L3 in the control variant (water).

**Figure 4:**
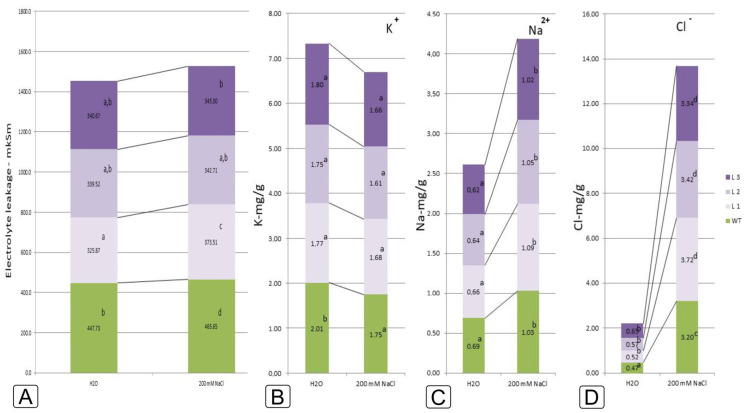
Characterization of the physiological and biochemical features of electrolyte leakage (**A**), the content of K^+^ (**B**), Na^+^ (**C**), and Cl^−^ (**D**) ions in response to 200 mM NaCl salinity in WT plants and *codA* transgenic tobacco plants. Different letters indicate significant differences between treatment, analyzed by a Duncan’s tests with the corresponding errors (*p* = 0.05).

Under salt stress in the leaves of tobacco plants of all variants of the experiment, the total content of electrolytes increased (by 1.01–1.15 times) and the content of potassium ions decreased (by 1.12–1.23 times) (Figure 4A). However, a significant difference in the increase in the content of electrolytes and the decrease in the content of potassium ions in the tissues of both control tobacco plants and plants of lines 1–3 was not revealed.

In Samsun tobacco plants and transgenic plants of lines 1–3 the different response to salt stress was clearly seen from the results of the content of sodium and chloride ions (Figure 4C,D). While in the leaves of the tobacco variety Samsun, the concentration of sodium increased by 1.9 times, and chloride ions by 6.8 times, in the leaves of transgenic tobacco plants of lines 1–3 this increase was significantly lower—sodium ions by 1.6–1.7 times, and chloride ions 5.1–6.2 times. It is known that salt stress can adversely affect plants by accumulating high concentrations of both Na^+^ and Cl^−^ ions, but the effects of the two ions may differ. A high concentration of Cl^−^ reduces photosynthesis and quantum yield due to the degradation of chlorophyll [20]. High Na^+^ content inhibits the uptake of K^+^ and Ca^2+^ ions, important plant nutrients, and impairs the effective regulation of stomata, resulting in reduced photosynthesis and growth.

Under salt stress, the K^+^/Na^+^ ratio decreased both in the control and experimental variants (Figure 4B,C). Maintaining a high K^+^/Na^+^ ratio is a defining characteristic of salt tolerance [21]. Samsun tobacco was found to be the most sensitive to salt stress, showing an imbalance in Na^+^ and K^+^ content, observed in the form of a lower K^+^/Na^+^ ratio (1.32). While in tobacco lines 1, 2 and 3 this ratio was 1.45; 1.44 and 1.43, respectively (Figure 4B,C).

When comparing the response to salt stress of *codA* transgenic lines 1, 2 and 3, it seems that L1 tobacco plants have the highest tolerance to salt stress, so they are able to maintain a lower rate of loss of potassium ions, which provides a favorable ratio of K^+^/Na^+^—2.68 in water culture and 1.45 at a dose of 200 mM NaCl. In line 2 tobacco the decrease in this ratio was from 2.73 to 1.44, and in L3 tobacco it was even more significant—from 2.95 in water culture to 1.43 under salt stress (Figure 4B,C).

**Figure 5:**
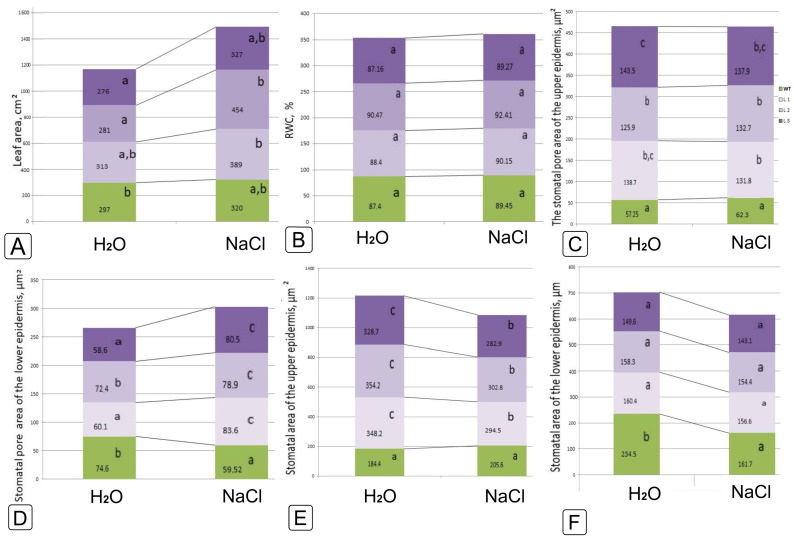
Characterization of the physiological and biochemical features of leaf area (**A**), hydration (**B**), size of stomatal pore (**C**,**D**) and stomatal guard cells (**E**,**F**) of upper (**C**,**E**) and lower (**D**,**F**) epidermis in response to 200 mM NaCl salinity in wild-type tobacco plants and *codA* transgenic lines. Different letters indicate significant differences between treatment, analyzed by a Duncan’s tests with the corresponding errors (*p* = 0.05).

The leaf area of the WT and *codA* transgenic lines slightly increased under the salinity impact, but there were significant differences only in the case of comparing the L2 line without salinity and under the application of 200 mM NaCl (Figure 5A).

Salinity had little effect on leaf water content (Figure 5B). The size of the stomatal opening of the upper and lower epidermis was found to be insensitive to this level of salinity in both WT plants and transgenic lines (Figure 5C,D). On the contrary, the stomatal area in WT plants decreased, while in transgenic lines it increased on the upper side of the leaf (Figure 5E). On the underside of the leaf, the area of stomata did not change in WT, while it decreased in transgenic lines (Figure 5F).

**Figure 6:**
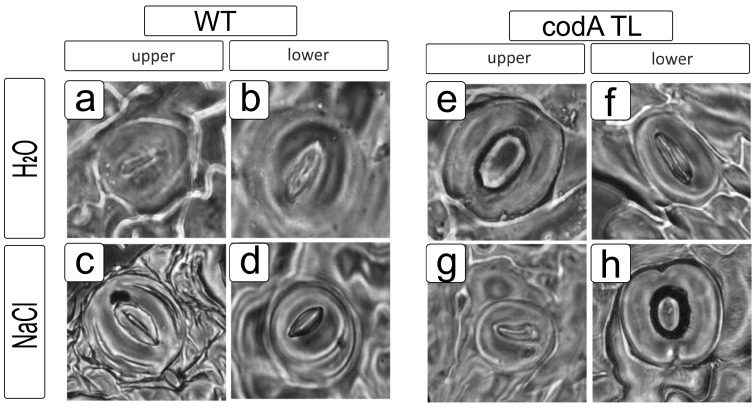
Morphological features of the stomatal apparatus of the leaf surface in response to 200 mM NaCl salinity in WT tobacco plants (**a**–**d**) and *codA* transgenic lines (**e**–**h**).

The shape of paired cells (guard cells) forming stomata and providing gas exchange by opening the aerenchyma cavity in WT plants was characteristic, providing the lenticular shape optimal for the regulation of the stomatal gap (Figure 6a–d). In transgenic plants, the stomata had a change in the inner wall and conjugation sites, leading to a change in shape manifested in forming an oval target (Figure 6e–h) rather than a lenticular shape. In both transgenic and control plants, the NaCl action caused asymmetry and differences in size between paired stomata (Figure 6c,d,g,h).

**Figure 7:**
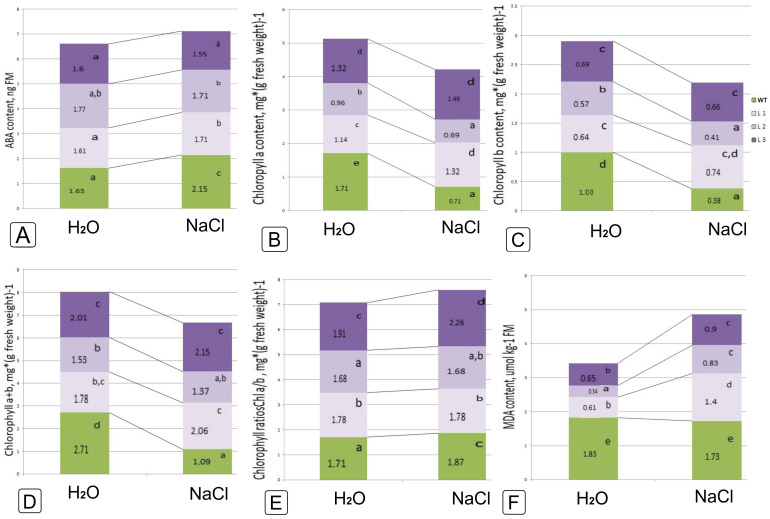
Characterization of the physiological and biochemical features of ABA (**A**), chlorophyll a and b (**B,C**), a + b (**D**) and their ratios (**E**), as well as MDA (**F**) in response to 200 mM NaCl salinity in wild-type tobacco plants and *codA* transgenic lines. Different letters indicate significant differences between treatment, analyzed by a Duncan’s tests with the corresponding errors (*p* = 0.05).

We registered a significant increase in ABA values under salinity both in the WT plants and in L1 and L3 transgenic lines, with the exception of L2, where the value did not change significantly (Figure 7A).

Salinity caused a significant decrease in the content of chlorophyll a in the control and somewhat less pronounced in line 2, while in transgenic lines L1 and L3 the values increased for chlorophyll a or remained unchanged for chlorophyll b (Figure 7B,C). It is even more noticeable when comparing the sum of chlorophyll (Figure 7D). When analyzing the ratio of chlorophylls under NaCl salinity, one can note an increase for WT and L3, which was not noted for L1 and L2 (Figure 7E).

The level of malondialdehyde did not change in WT plants, but increased in *codA* transgenic lines, at the same time, it did not reach control values at a given salinity intensity and remained lower than in WT (Figure 7F).

**Figure 8:**
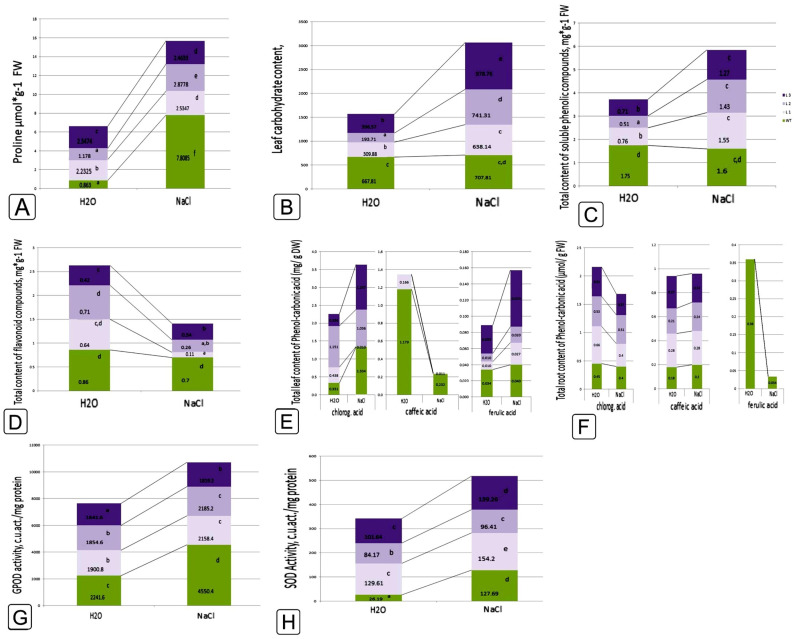
Characterization of the physiological and biochemical features of proline (**A**) free sugar (**B**), phenolic compounds (**C**), flavonoids (**D**) and phenol-carbonic acids (chloric, caffeic and ferulic) in leaf (**E**) and root (**F**), as well as GPOD (**G**) and SOD (**H**) activity in response to 200 mM NaCl salinity in WT tobacco plants and *codA* transgenic lines. Different letters indicate significant differences between treatment, analyzed by a Duncan’s tests with the corresponding errors (*p* = 0.05).

Salinity caused a significant increase in the level of proline in both WT and transgenic lines; however, if in transgenic lines the maximum growth was 2.5 times, then in WT plants a 10-fold increase in this indicator was marked (Figure 8A). Meanwhile, NaCl did not affect the amount of sugars in WT plants, while the pool of sugars increased significantly in transgenic lines (Figure 8B).

The total amount of phenolic compounds and flavonoids in WT did not depend on the effect of salt (Figure 8C,D). At the same time, salinity caused an increase in phenolic compounds in *codA* overexpressing plants (Figure 8C). The amount of flavonoids in the transgenic lines was significantly reduced (Figure 8D). In the analysis of phenolic acids, it was found that the amount of chloric acid somewhat decreased under salinity, while the amount of coffeic acid increased, both in WT plants and in plants overexpressing *codA*. However, differences between WT and *codA* transgenic lines persisted (Figure 8E,F). Also, in WT plants, there was a significant drop in the amount of ferulic acid that was absent in transgenic lines.

The response of the activity of the antioxidant enzymes SOD and GPOD to salinity was the same for transgenic and non-transgenic plants. There was a significant increase in relation to the indicators in the absence of NaCl. However, the increase in growth activity in WT plants was more significant (Figure 8G,H).

**Figure 9:**
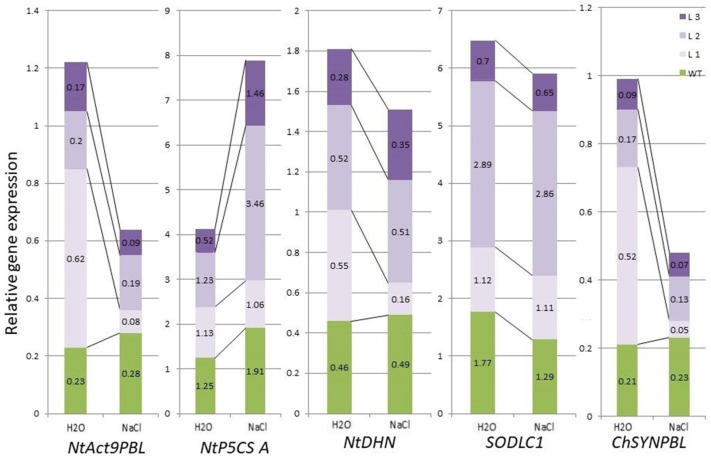
Peculiarities of *NtAct9PBL*, *NtP5CS A*, *NtDHN*, *SODLC1*, *ChSYNPBL* gene expression in leaves in response to 200 mM NaCl salinity in wild-type (WT) tobacco plants and *codA* transgenic lines with constitutive expression of choline oxidase.

To assess the effect of endogenous increase in glycine betaine and choline oxidase expression-mediated consequences, we selectively assessed the gene expression level in WT plants and *codA* transgenic lines under normal conditions (watering) and application of 200 mM NaCl salinity (Figure 9). Four genes encoding the following proteins were chosen: Δ′-pyrroline-5-carboxylate synthetase (NtP5CS), which is responsible for proline synthesis; dehydrine (NtDHN), responsible for protective reactions to dehydration; Cu/Zn superoxide dismutase (SODLC1), which is involved in defense against oxidative stress and chalcone synthase (ChSYNPBL), as a key enzyme of flavonoid derivatives production. Only the expression of the *NtP5CS* gene was found to be up-regulated in the *codA* transgenic tobacco lines when exposed to NaCl, especially in transgenic lines L2 and L3 (Figure 9). In WT plants, the expression of the *NtP5CS* gene also increased. It was found that while the expression of the *NtDHN* and *SODLC1* genes was slightly down-regulated in *codA* transgenic tobacco lines, the expression of the *ChSYNPBL* gene was quite noticeably reduced under NaCl exposure (Figure 9).

**Figure 10:**
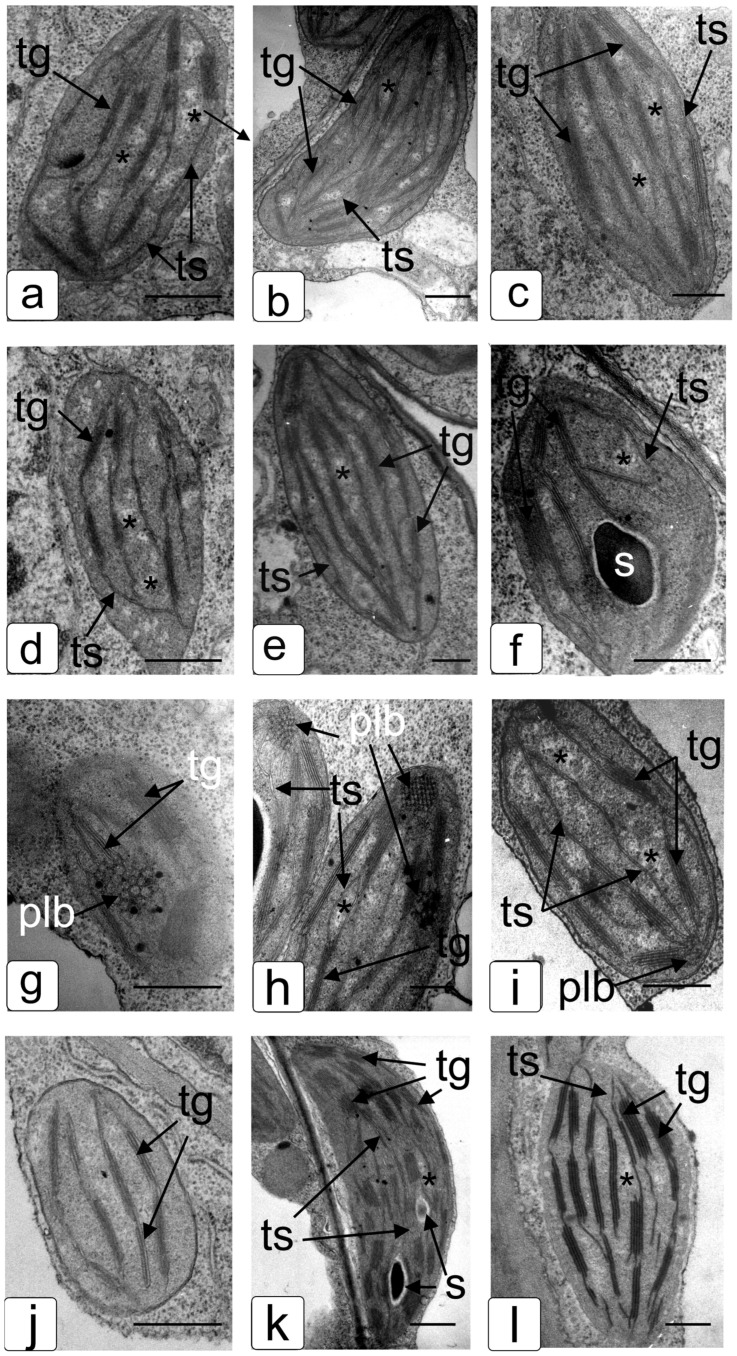
Ultrastructure of plastids mesophyll cells in the third tobacco cv. Samcun leaf (**a**–**f**) and *codA* transgenic line L2 (**g**–**l**). The plants were cultivated in pot culture in the absence (**a**–**c**,**g**–**i**) of salt application and under the watering of 200 mM NaCl (**d**–**f**,**j**–**l**). Typical ultrastructural images of plastids in satellite cells of small mesophyll vessels (**a**,**d**,**g**,**j**), columnar (**b**,**e**,**h**,**k**) and spongy (**c**,**f**,**i**,**l**) parenchyma cells are shown. Abbreviations: plb—prolamellar bodies; tg—thylakoids of grana; ts—thylakoids of stroma; s—starch; *—nucleoid. Scale bar: 5 μm.

Ultrastructural analysis of leaf chloroplasts in WT tobacco plants revealed formations characteristic of photosynthetic tissue (Figure 10). In the plastid stroma, fragments of a branched lamellar structure evenly distributed throughout the volume can be observed (Figure 10a–c). There are fragments of granal complexes of thylakoids arranged in stacks, which are connected with the thylakoids of the stroma with the help of lamellar formations. Between which there are light areas of the stroma, characteristic of the localization of nucleoids. The lenticular shape of the plastids was typical for these tissue types of the newly formed leaves; therefore, there was no large number of plastoglobules and other inclusions characteristic of more mature tissue. The influence of salinity did not significantly affect the shape of plastids in cells of WT plants (Figure 10d–f). However, one can note a decrease in the number of granae and thylakoids in a grana, a decrease in the number of lamellar formations in the chloroplasts of spongy and columnar parenchyma, and a decrease in the number of light areas of nucleoids per plastid section (Figure 10e,f). We do not notice significant deposits of starch, which is associated with fixation in the morning. Chloroplasts of *codA* transgenic plants also had a round-lenticular shape; however, in addition to the granal and lamellar system of thylakoids, the prolamellar bodies characteristic of developing (greening) plastids, which are inherent in plastids of young or etiolated plants transferred from darkness to light were observed (Figure 10g–i). Prolamellar bodies were noted in all tissues, which probably explains the delay in the formation of membrane complexes in photosystems. 

The plastids of the columnar parenchyma were more developed, although they had a large number of nucleoids and prolamellar bodies (Figure 10h), while the plastids of the underlying spongy parenchyma had a more pronounced developmental delay and many light areas in the stroma (Figure 10i). The NaCl treatment led to the formation of chloroplasts with a reduced number of nucleoids and the absence of prolamellar formations (Figure 10j–l). Evenly spaced granae, a network of granal and lamellar thylakoids, and a small amount of nucleoids indicated the completion of chloroplast formation in the columnar and spongy mesophyll (Figure 10k,l). More contrasting membranes probably indicate a greater incorporation of protein molecules related to photosynthetic complexes. Also, the characteristic smaller size and ultrastructure of the companion cell plastid corresponded to ones typical of developed mesophyll tissue of tobacco cells.

## 3. Discussion

Compatible solutes, being low molecular weight soluble compounds, are usually non-toxic at high concentrations. It has been shown that an increase in their concentration protects plants from stress damage through adaptation to damage by detoxifying reactive oxygen species, protecting membrane integrity and stabilizing enzymes/proteins [22]. They also protect cellular components from disruption of turgor and damage during dehydration of cellular compartments, acting as osmoprotectants. Such solutes include compounds formed during primary and less commonly secondary metabolism and are represented by proline, sucrose, polyols, trehalose, and glycine betaine [1]. There are several strategies for studying the role of such compounds, including exogenous and endogenous regulation of the response to stress [23]. A wide range of work on understanding the functional role and application of these substances to prevent stress damage in glycophyte plants includes both strategies [24]. At the same time, the study of the functioning of the endogenous pool of this osmolyte allows a more complete assessment of the prospects for its engineering [25]. It is currently believed that the protective effect of glycine betaine is based not only on the action of the substance itself but also on its concomitant effect on the induction of the expression of other genes involved in increasing resistance and triggering the synthesis of the corresponding products [26].

Glycine betaine is found at very low levels in tobacco and did not increase appreciably when exposed to stress factors [27]. We used previously obtained transgenic plants with a construct containing an enzyme that allows obtaining glycine betaine not in a typical way for plants (for example, a sequential conversion of several steps [25]), but with the help of the bacterial choline oxidase enzyme from *Arthrobacter globiformis* (allowing the conversion of choline to glycine betaine in one step [28], which showed high tolerance to NaCl salinity in callus culture [19]. Transgenic plants expressing the *codA* gene when cultivated without sodium chloride were smaller and lighter, although the size of the leaves and their water content did not differ, which indicates a change in photosynthetic activity and a likely slowdown in development. It was also noted that, unlike WT plants, which formed inflorescences and passed into the generative development stage, transgenic plants were in the previous stage of active vegetative growth. This result can be explained if we consider it from the point of view of a possible connection with the regulation of phytohormones [29]. It has been shown that glycine betaine biosynthesis provokes changes in ethylene synthesis and increases the expression of auxin-responsive gene levels [30]. In the works of other authors devoted to transgenic plants, growth retardation and color change in the absence of stress factors were not noted.

Changes in the size of the stomatal pore (stoma) and stomata indicate a significant effect of endogenous glycine betaine on the formation of the system of regulation of air exchange, respiration, and transpiration, which should probably ensure the preservation of moisture in the leaves at the same level as in the control, despite differences in size. Thus, he foliar application of glycine betaine has been shown to improve growth, upregulate osmoprotection and osmoregulation, increase relative water content, net photosynthetic rate, and catalase activity, decrease photorespiration, ion leakage, and lipid peroxidation, protect the oxygen-evolving complex, and prevent chlorosis [31]. Probably, it is this feature that makes it possible to maintain the hydration of tissues during drought, salinity and water stress [32,33,34]. In addition, glycine betaine is able to significantly (three times) improve stomatal conduction [33].

Under favorable growing conditions, the levels of ABA in the transgenic and WT plants were identical, suggesting that gene expression and the appearance of glycine betaine themselves did not cause stress in tobacco plants. It is known that the formation of GB, as well as its further accumulation, is genotype-specific. This means that for the reaction of all metabolic chains, plants do not depend on the direct action of GB [35]. Typical plant systems that accumulate GB are associated with the genes responsible for the accumulation of the precursor through glycine in chloroplasts [36]. In this work (as in many others), the signal sequence of the rubisco small subunit gene [18] was used to deliver the enzyme to the substrate choline, which is formed in the chloroplasts of leaf cells of plant systems. Accordingly, the product of interest, GB, is also synthesized there.

Based on changes in GB translocation within the plant, some authors suggest that GB translocation occurs from older parts of plant systems to new ones in order to protect those [25]. It is possible that when comparing identical, already-formed leaves (we analyzed three fully developed leaves), differences will not be found.

The indicators of the content of chlorophyll a and b and the ratio between them, as expected from the noted differences in the color of foliage, indicated the development inhibition of the photosynthetic apparatus in transgenic plants. A similar effect has been described for the exogenous action of GB on tea leaves. The authors state that spraying GB regulated etioplast–chloroplast transition in leaf cells, significantly increased epigallocatechin gallate, theanine, and caffeine contents, and lowered chlorophyll content in albinistic young leaves of WL-1 tea variety, thus improving its quality in some aspects, maintaining special leaf color, exerting flavor and umami, and this is accompanied by improving and refreshing effects [37]. This is partly confirmed by the low level of MDA found in this work compared to WT under control conditions. A decrease in the MDA level is a characteristic feature of the lines we obtained [38]. Similar effects were observed both with exogenous application of GB [39,40,41] and with an endogenous increase in the content of GB due to transgenesis [42].

Glycine betaine and proline are the most common osmolytes under stress conditions [43]. Two transgenic lines (L1 and L3) had a significantly larger proline pool, which is possibly due to the synergistic effect of the influence of two osmotically active substances and their mutually mediated influence on the switching-on of genes associated with the response to stress, which were also induced in the absence of a stress factor [44]. Most likely, this is a consequence of ectopic expression of the *codA* gene due to the constitutive 35S promoter.

A reduced amount of flavonoids and phenolic compounds in transgenic lines also characterized these plants as conditionally “more mature” [45], which contradicted a less intense green color and was probably associated with the peculiarities of biosynthesis and outflow of secondary metabolites in transgenic plants. However, this did not prevent further stress protection and, thus, was consistent with the data obtained both with exogenous application and with an endogenous increase in the content of GB [46].

A significant increase in the pool of chlorogenic and caffeic acids in transgenic plants expressing *codA* was possibly induced by the acceleration of biosynthesis, and a decrease in the content of ferulic acid can be caused both by its consumption and by the cessation of its biosynthesis from caffeic acid, which can partially explain the increase in the pool of the latter in transgenic lines. It is possible that this may be reflected in the features of cell wall lignification. Modulation of organic acid scores has been described previously [47].

The noted increase in the activity of superoxide dismutases in transgenic plants may be associated with the influence of GB. Such an effect of GB on SOD activity was described in a number of studies on its osmoprotective and anti-stress properties [40,48,49,50].

In general, plants with overexpression of the *codA* gene were characterized by a number of physiological features of the metabolic processes of primary and secondary metabolism, such as the amount of sugars, the activity of antioxidant enzymes, flavonoids, and phenolic acids. Assuming that such changes may provide transgenic plants with additional opportunities to withstand abiotic stresses, we studied these parameters when the plants were exposed to NaCl. For a number of parameters studied, the differences between transgenic lines and WT plants were also significant: upon exposure to 200 mM NaCl for two weeks, transgenic plants expressing *codA* passed to the generative phase of development and formed flowering organs; most of the leaves of *codA* transgenic tobacco plants during this time turned dark green without features of the development inhibition, noted in the absence of salt stress (Figure 1 and Figure 2). After three days of watering with 200 mM NaCl solution, *codA* transgenic plants (L1, L3, L3) showed normal development and growth characteristics of WT plants in the absence of salinity (Figure 2). Thus, the appearance and physiological state of *codA* transgenic lines looked better in the presence of a stress factor (NaCl) than without it. The synthesis of glycine betaine gives an advantage exactly under salinity, and under normal conditions (without salinity) does not provide benefits. In contrast to the WT plants, the *codA* transgenic plants developed more slowly and their photosynthesis was weaker in the absence of salt stress. This disadvantage was fully compensated by salinity.

Morpho-anatomical characteristics of leaves, as well as the size and opening of stomata, are important factors affecting plant stress tolerance and the efficiency of water use and CO_2_ uptake since the stoma area positively correlates with transpiration [51]. Stomata closure is the first response of plants to salinity by limiting transpiration, resulting in reduced stomatal conductance [52]. ABA directly affects ion transport in guard cells, rapidly changing stomatal pores in response to changes in water availability and abiotic stresses [53]. It was shown that the ABA content in *codA* transgenic plants almost did not change after salt treatment (Figure 7A), while the area of the stomata pores both on the upper and lower epidermis decreased by more than two times (Figure 5C,D). In WT plants, although the amount of ABA increased under salinity, the area of stomata changed differently in the upper and lower epidermis: it decreased in the stomata of the lower epidermis (Figure 5D,F) and slightly increased in the stomata of the upper epidermis (Figure 5C,E). Thus, not only ABA changes the opening of the stomatal opening, but also some other factors, which can be the accumulation of osmotic agents or the activation of certain genes under stress [54].

The total content of chlorophyll (Chla + Chlb) in WT plants decreased by almost 2.5 times under salinity (Figure 7D), while in two transgenic lines, it increased (Line 1 and 2), and in Line 3 it decreased by 1.39 times (Figure 7D). At the same time, the difference between the content of chlorophyll a and b in the transgene lines remains constant under salt stress, while it increased in WT and line 3 (Figure 7B,C). Apparently, Lines 1 and 2 were more resistant to stress conditions than WT plants and transgenic Line 3. Similar changes in chlorophyll content were observed in some plant species subjected to salt stress, which may be associated with impaired biosynthesis or accelerated pigment degradation [55,56,57].

Peroxidation of polyunsaturated fatty acids in the plasma membrane results in the formation of malondialdehyde (MDA), which leads to a decrease in cell membrane fluidity [58]. Hence, the content of MDA in plant tissues can be an indicator of oxidative damage [59]. Our study revealed differences in lipid peroxidation levels between WT plants and *codA* transgenic lines when treated with 200 mM NaCl (Figure 7F). In WT plants, MDA content remained virtually constant after salt exposure, while transgenic plants showed dialdehyde accumulation similar to the results by Marċek et al. (2014) [60]. This may indicate that these tobacco lines are becoming more salt tolerant in some other way than increasing cell membrane stability.

To cope with salt stress, plants increase the osmotic potential of their cells by synthesizing and accumulating compatible osmolytes such as proline and glycine betaine, which are involved in osmotic adaptation [61]. In our study, the proline content under the influence of NaCl significantly increased in WT plants, increasing by 800%, while in *codA* transgenic lines 1 and 3, its content remained almost at the same level, and in line 2, its content increased only by 200% (Figure 8A). Salt tolerance was higher in *codA* transgenic plants than in WT plants. The accumulation of glycine betaine in *codA* transgenic lines had a positive effect on the induction of other protective factors, which indirectly caused a dramatic increase in proline content. Similar results were obtained by Goel et al. (2011) with *codA* transgenic tomatoes, in which the level of proline contained in unstressed plants was higher than in WT plants [13].

Accumulating carbohydrates in plants also plays an important role in stress mitigation, including osmoprotection, carbon storage, and removal of reactive oxygen species [62]. Soluble sugars, photosynthetic products (glucose, fructose, and sucrose), interacting with phytohormones, play an important role in maintaining the overall cellular structure, in growth and development, and in the complex regulation of stress responses [63,64,65,66]. It was shown that the amount of carbohydrates in WT plants did not change when exposed to salt, while in *codA* transgenic plants, the content of soluble sugars increased; however, changing by 2 times in L1, 3.8 times in L2, and 2.5 times, respectively, in L3 (Figure 8B). The data obtained in different studies vary greatly depending on the plant genotype. For example, Boriboonkaset et al. (2012) observed that the total soluble sugar content in the flag leaf of a salt-sensitive rice variety subjected to salt stress increased significantly [67], but Mišiċ et al. (2012), when studying *Schenkia spicata* plants, showed that sugar levels in the salt-sensitive genotype did not change under salt stress [68]. When tomato plants (*Solanum lycopersicum* L.) were treated with 100 mM NaCl, the levels of sucrose and glucose decreased both in the leaves and in the roots [69].

Under stressful conditions, the synthesis of phenolic compounds is activated in plants, protecting the launch of antioxidant defenses through alteration of metabolic pathways, and the enzymes involved [70]. Phenolic compounds are involved in the regulation of plant growth, and the functional activity of chloroplasts and mitochondria, as well as protection against stress effects due to interaction with reactive oxygen species [71]. If the initial level of phenolic compounds was quite high when exposed to NaCl on WT of tobacco plants, then in *codA* transgenic lines with a low amount of phenolic metabolites, their number increased significantly (Figure 8C). This indicates the activation of the accumulation of these bioantioxidants, the amount of which increases under stressful conditions [72]. The content of flavonoids in tobacco leaves of all evaluated variants after exposure to NaCl decreased: in line 3 and control—by 20%, in line 2—by 60%, and in line 1—by 80% relative to the stress-free variant (Figure 8D). In the absence of stress, the amount of flavonoids in transgenic lines was lower than in the original variant. Their number on the seventh day of exposure to NaCl decreased significantly in transgenic plants (Figure 8D). Apparently, this occurs as a result of the fact that flavonoids are antioxidant metabolites, which are the first to participate in the protection of plants from the action of ROS [73]. Our next task was to study the accumulation of such compounds of the phenylpropanoid pathway as caffeic acid, which serves as a precursor in the formation of ferulic acid, as well as chlorogenic (5-caffeyl-quinic) acid. The accumulation of ferulic acid was noted only in the leaves of plants of the control variant. In the absence of stress exposure, its level was 0.36 µM/FW, while under stress it reached 0.034 µM/FW, that is, it decreased by almost 10 times (Figure 8E,F). These changes, apparently, are a consequence of the active use of ferulic acid as a precursor for the biosynthesis of other phenolic metabolites in stressed plants.

The caffeic acid content in the leaves of tobacco plants of all studied variants was 0.18–0.28 µM/FW and did not change under stress (Figure 8E,F). Its lower level was in the control variant and line 2, while in lines 1 and 3 it was 40% higher. Consequently, in the leaves of control and transgenic tobacco plants, stress conditions did not cause changes in the accumulation of caffeic acid. The accumulation of chlorogenic acid in the leaves of tobacco plants of all evaluated variants was 1.5–2 times higher than the content of its precursor, caffeic acid. This was more characteristic of *codA* transgenic lines. In response to NaCl stress, the content of chlorogenic acid did not change in plants of the control variant and line 2, while in lines 1 and 3 it decreased (by 34% and 40%, respectively) (Figure 8E,F). This once again indicated some differences in the phenolic compounds biosynthesis in *codA* transgenic tobacco plants.

Antioxidant enzymes are also involved in the first line of plant cell defense against ROS. Superoxide dismutase (SOD) is the main scavenger of superoxide O_2·_^−^ and its enzymatic function results in the formation of H_2_O_2_ and O_2_. The resulting hydrogen peroxide is then neutralized by various peroxidases (PODs), in particular, guaiacol peroxidase (GPOX). In our work, we showed that NaCl treatment in WT plants caused a significant increase in proline content and, at the same time, there was an increase in superoxide dismutase activity by almost 6 times, and GPOX activity by 2 times (Figure 8G,H), which coincides with the results presented in Hoque et al. (2008) [74]. At the same time, the activity of these enzymes in *codA* transgenic lines did not actually change (Figure 8G,H). The activity of antioxidant enzymes, such as catalase (CAT), ascorbate peroxidase (APX), guaiacol peroxidase (POD), glutathione reductase (GR) and superoxide dismutase, increases under salt stress in plants and correlates with salt tolerance [75,76,77,78]. The activity of these enzymes can be well preserved in the glycine betaine presence. Accordingly, these antioxidant enzymes can effectively scavenge active oxygen and oxygen free radicals and thus maintain the structural stability and integrity of the cell membrane and chloroplast membrane under stress conditions [7].

When comparing the response to salt stress of transgenic plants in comparison with the original WT plants, a change in the processes of primary and secondary metabolism was revealed, related to the redox balance of the cell, osmotic characteristics inside the cell, as well as the synthesis of a number of such secondary metabolites as phenolic compounds, flavonoids and phenolic (phenol-carbonic) acids (Figure 8). Biosynthesis changes can occur both at the level of activation/deactivation of existing processes (due to specific or non-specific regulation of enzymatic processes) [79] and at the level of induction of responsive gene expression associated with these processes [80,81]. To explore possible concomitant responses of other genes associated with the abiotic stress response, the expression levels of genes involved in responses to abiotic stress were assessed in *codA* transgenic tobacco plants under normal and NaCl stress conditions. Four genes encoding the following proteins were chosen: Δ′-pyrroline-5-carboxylate synthetase (NtP5CS), which is responsible for proline synthesis; dehydrine (NtDHN), responsible for protective reactions to dehydration; Cu/Zn superoxide dismutase (SODLC1), which is involved in defense against oxidative stress and chalcone synthase (ChSYNPBL), as a key enzyme of flavonoid derivatives production [82,83]. The data obtained indicate that, as expected, the expression of the *NtP5CS*, *NtDHN*, *SODLC1*, and *ChSYNPBL* genes in WT plants changed under salinity. This confirmed their involvement in the stress response. On the contrary, significant changes in metabolism in *codA* transgenic lines were demonstrated. If no change in the expression of *SODLC1* was observed in all three *codA* transgenic lines, then a difference in the expression of the *NtP5CSA*, *NtDHN*, and *ChSYNPBL* genes can be noted between in L1 and in L2 and L3 lines (Figure 9). Thus, in L1, the *NtP5CSA* expression did not change, while the expression of the *NtDHN* gene was down-regulated, as was the expression of the *ChSYNPBL* gene during NaCl exposure. In two other lines, L2 and L3, there was a threefold increase in the expression of the *NtP5CSA* gene. The expression of both the *NtDHN* gene and the *ChSYNPBL* gene did not change. Thus, it can be noted that transgenic plants significantly differed from WT plants in response to NaCl. Although, they significantly differed from each other. To understand this phenomenon, it seems important to further study a wider pool of genes related to the response to stress. Surprisingly, the expression of the housekeeping gene *Act9*, which is often used as a reference gene, was significantly changed in the L1 and L3 transgenic lines (Figure 9). It can be assumed that a reference gene suitable for one experiment may not be suitable for another experiment [84]. Although the level of expression varies depending on different stress conditions and developmental stages, some housekeeping genes show variability in different plant organs [85]. When conducting a correlation analysis, differences in the degree of correlation and differences between the lines were revealed, while a number of correlations had high rates both positive and negative (Figure S8). It can be assumed that in order to identify more pronounced patterns, it would be correct to expand the pool of studied genes since the established patterns should be reflected both in the upstream processes (at the level of the expression regulation of transcription factors and various kinases) and in the downstream (dependent) processes of regulation of codependent and sensitive genes [86].

During the formation of photosynthetic structures under sufficient lighting conditions, a rapid transformation of prolamellar bodies into stromal lamellae and granal system lamellae occurs [87]. The delay in the development of the membrane structure of plastids was characterized by a change in the nucleoid and a qualitative and quantitative delay in the development of the photosynthetic infrastructure [88]. The presence of prolamellar bodies in the leaf mesophyll cells of transgenic tobacco plants grown under normal conditions (Figure 10g–i) and, to a less pronounced extent, in the cells of WT plants in the presence of NaCl (Figure 10f) indicates a slowdown in the process of de-etiolation and the formation of full-fledged structures providing both stages of photosynthesis. When exposed to salinity, this effect completely disappeared in *codA* transgenic plants (Figure 10j–l).

## 4. Materials and Methods

### 4.1. Plant Material and General Growth Conditions

Tobacco plants (*Nicotiana tabacum* L.) cv. Samsun, transformed as described earlier [19] using *Agrobacterium* strain AGL0 with a genetic construct containing the choline oxidase synthesis gene from *Arthrobacter globiformis* (*codA*) (Figure S9). The presence of transgene in plants has been proven by PCR. The expression of the *codA* gene in transgenic plants was shown by RT-PCR earlier [19]. In vitro, plantlets were propagated by cuttings on the modified Murashige–Skoog (MS) basal medium (½ macrosalts, full composition of microsalts and iron chelate without plant growth regulators, supplemented with 0.7% sucrose and 0.7% agar-agar) and maintained in the phytotron chamber with 16/8 h (day/night) photoperiod at an illumination intensity of 250 µM m^−2^ s^−1^ and temperatures of 24–25/19–20 °C (day/night). Before adapting to the soil conditions (commercial universal soil mixed with perlite 3:1), in vitro, plantlets were soaked in sterile distilled water and transferred to the soil in a day. Treatment with salinity was carried out after the formation of 8–9 leaves in plants by watering the soil in pots with 200 mM NaCl solution. The solution amount was added by calculating the soil moisture capacity in the pot vessel by gravimetric method.

Wa = (Mv − Ms):Ms × 100%,

where

Wa is soil moisture;
Mv is the weight of wet soil;
Ms is the weight of dried soil.

Control plants were watered with distilled water. Soil moisture was maintained at 70% and controlled by weighing each pot vessel during growth.

Three independent transgenic lines expressing the *codA* transgene were used. Untransformed wild-type (WT) Samsun tobacco plants were also maintained both in vitro and in pot culture as a control. In the experiments, five plants of each line were used. Samples were taken after 7 days using mixed plant material of three medium leaves (9, 10, and 11 plant leaves). Samples were weighed and frozen in liquid nitrogen and stored at −70 °C until they were required for analysis.

### 4.2. Determination of Glycine Betaine Content

Measurement of GB was performed according to [89]. Fresh leaves (1 g) were mechanically shaken with 20 mL of deionized water for 48 h at 25 °C. The samples were filtered and the filtrates were diluted (1:1) with 2N H_2_SO_4_. Aliquots (0.5 mL) were transferred to centrifuge tubes and incubated in ice water for 1 h. This was followed by adding 2 mL of cold potassium iodide-iodine reagent, gently mixed, and the tubes were stored at 48 °C for 16 h. After centrifugation at 10,000× *g* for 15 min at 8 °C, the supernatant was carefully aspirated. The per iodide crystals were dissolved in 9.0 mL of 1,2-dichloroethane, and the absorbance was recorded spectrometrically at a wavelength of 365 nm. The reference standards of GB (50–200 mg ML^−1^) were prepared using 1N H_2_SO_4_ [90].

### 4.3. Ions Uptake Determination

For determining the ion contents (K^+^, Na^+^, Cl^−^) in tabaco WT and transgenic Lines tissues, the elements were obtained from 200 mg fresh weight of leaves in 25 mL of deionized water for 30 min at 40 °C for ultrasonic extraction (Sapphire, Moscow, Russia). The ultrasound power level was 35 kHz. The samples were cooled and passed through a porous filter (0.45 µm). The determination of the content of the elements in the samples of leaves extracts was carried out using ion-selective electrodes: ELIT-031—potassium electrode; ELIS-112—sodium electrode; ELIT-261—chloride electrode using ITAN ionometer (TomskAnalit, Tomsk, Russia). The ions content in mg L^−1^ was obtained by plotting the dependence of the EMF in the concentration of ions according to a pre-built scale of reference solutions in the studied concentration range (10^−2^–10^−5^ M). A silver chloride electrode was used as a reference electrode (EVL-1M3.1) (GZIP, Gomel, Belarus). The content of electrolytes in plant tissue samples was measured by the electrical conductivity of the solution using a conductometer Expert-002 (Ekoniks, Moscow, Russia). The change in the electrical conductivity of the samples in µS g^−1^ is proportional to the concentration of electrolytes and equivalent to the degree of accumulation of ions in plant tissues. The electrolyte contents in tobacco WT and transgenic lines tissue samples were measured by the electrical conductivity of the solution using a conductometer Expert-002 (Ekoniks, Moscow, Russia). The change in the electrical conductivity of the samples in µS g^−1^ is proportional to the concentration of electrolytes and equivalent to the degree of accumulation of ions in plant tissues [91,92].

### 4.4. Water Content Measurement

To measure water content, leaf segments were weighed (three replicates) on an analytical balance and placed in glass bottles of known mass. The bottles with leaves were transferred to a thermostat at 95 °C for 2 h, then the temperature was lowered to 65 °C and the leaves were left for a day to dry to a constant weight. The dry weight of the sheet was determined by the difference between the weight of the bottle with the sheet and the weight of the empty bottle. Next, the water content of the leaf was calculated using the formula: (m FW leaf − m DW leaf)/m FW leaf) 100%. Water content is expressed as a percentage of the water content in the leaf.

### 4.5. Measurement of Leaf Area

Selected leaves of the middle tier were measured using the planimetric method of counting squares according to Beerling et al. (1990) [93].

### 4.6. Study of the Stomatal Apparatus

For a comparative study of the stomata apparatus, the middle third of plant leaves was used; for each variant, at least 100 cells obtained from fragments of three independent plants were analyzed. Replicas (imprints) of the surface of the leaf blade were obtained by applying colorless nail polish to fresh plants with a brush. After the varnish had dried, the film replicas were separated from the leaves, glued onto a glass slide, and used for microscopy and photomicrography. The replicas were analyzed using a microscope (Olimpus with a Canon A640 photo attachment (10 MegaPixels), ×40 objective) (Shinjuku, Tokyo, Japan). Digital micrographs of the leaf surface were analyzed by Digital Micrograph software (GMS 3, Gatan, Pleasanton, CA, USA). Statistical treatments of experimental data were performed at a 5% significance level using the analysis of variance (ANOVA) and Duncan’s multiple range tests with AGROS software (version 2.11) (AGROS, Moscow, Russia), as well as standard MS Excel 2010 software packages (One Microsoft Way, Redmont, WA, USA).

### 4.7. Determination of Pigments

Extraction of pigments from leaves was carried out with 96% ethanol [94]. The leaves of the plant (0.1 g of fresh weight) were placed in volumetric test tubes and filled with 4 mL of ethanol. To determine chlorophyll, leaf tissue was ground in a mortar with the addition of a small amount of zeolite. The homogenate was centrifuged at 10,000 rpm at 4 °C for 10 min. The absorption of the supernatant was measured at 649, 665 nm on a Genesis 20 spectrophotometer (ThermoSpectronic, Sandy Spring, MD, USA), and the content of pigments was calculated by the formula:

C_chl a_ = 13.70 D665 − 5.76 D649;

C_chl b_ = 25.80 D649 − 7.60 D665;

where

chl a is chlorophyll a, chl b is chlorophyll b.

### 4.8. Lipid Peroxidation

Peroxidation of lipids in plant leaves was analyzed by the content of malondialdehyde (MDA) determined in the reaction with thiobarbituric acid (TBA) [95]. A sample of plant tissue weighing 0.2 g was homogenized homogenized in 10 mL of 0.1 mol L^−1^ TRIS-HCl buffer with pH 7.6 containing 0.35 mol L^−1^ NaCl. To 3 mL of the homogenate was added 2 mL of 0.5% TBA in 20% TCA. The resulting mixture was heated at 100 °C for 30 min, quickly cooled, and filtered [96]. The optical density of the filtrates was measured at 532 and 600 nm. The MDA concentration was calculated using the molar extinction coefficient equal to 1.56 × 10^–5^ M^–1^ cm^–1^. The analysis was performed in three repetitions and the concentration was calculated by the formula

C = (E532 − E600) × n/(ε × l × m),

where

C is the concentration of MDA, µmol MDA g^−1^ fresh weight
E—optical density of the solution
n—dilution
ε—Coefficient of molar extinction MDA (156 mM^−1^·cm^−1^)
l—The length of the optical path of the light beam, cm.

### 4.9. Determination of SOD Activity

Determination of SOD activity was carried out according to the method of Beauchamp, Fridovich (1971) [97]; 0.2 g of plant material was triturated in liquid nitrogen. Extraction of soluble proteins was performed with 0.066 M K/Na-phosphate buffer, pH 7.4, containing 1 mM dithiothriitol (DTT), 0.5 mM phenylmethylsulfonyl fluoride (PMSF) in DMSO, 1–3 mg polyvinylpyrrolidone (PVP). The mixture was centrifuged at 10,000× *g* for 20 min at 4 °C. The resulting supernatant was used as an enzyme preparation to determine the activity of SOD, which was judged by the inhibition of photoreduction of nitrobluetetrazolium (NBT). The incubation medium for determining SOD activity contained 50 mM K, Na-phosphate buffer (pH 7.8), 172 μM NBT, 210 μM methionine, 24 μM riboflavin, and 0.1% Triton X-100. SOD activity was measured by the decrease in optical density at 560 nm after 30 min of incubation on a Genesis-10 UV spectrophotometer. SOD activity was expressed in conventional units per 1 mg of protein in 30 min (conventional unit/mg of protein). The reaction was carried out under illumination with fluorescent lamps model F36W/54 “Philips” for 30 min. Enzyme activity was calculated using the formula:

A = (lg(E_contr_/E_exper_)/(lgn × Valiq × C),

where

A—SOD activity, c.u./mg of protein
E_contr_—optical density of the control sample
E_exper_—optical density of the experimental sample
n—reaction mixture volume, mL
V_aliq_—volume of enzyme aliquot, mL
C—concentration of fresh biomass in the enzyme extract, mg.

### 4.10. Determination of Peroxidase Activity

Determination of peroxidase activity was carried out according to the method proposed by Ridge, Osborne (1971) [98] using guaiacol; 0.2 g of plant material was triturated in liquid nitrogen. Then, 0.066 M K/Na-phosphate buffer, pH 6.7, containing 1 mM dithiothreitol (DTT), 0.5 mM phenylmethylsulfonyl fluoride (PMSF) in DMSO, and 1 mg polyvinylpyrrolidone (PVP) was added to the homogenate. Peroxidase activity was measured using 7 mM guaiacol as a hydrogen donor and the assay was based on monitoring the rate of H_2_O_2_ interaction with POX. The optical density was measured in the first minute and the second minute after the addition of H_2_O_2_ on a Genesis-10 UV spectrophotometer (Thermo Electron Corporation, Waltham, MA, USA) with a wavelength of 440 nm. Enzyme activity was calculated using the formula:

A = ΔE × n/ε × W × l

where

A—enzyme activity, µmol guaiacol/mg biomass × min
ΔE—average optical density per minute (t2 − t1)/2
n—dilution
W—wet tissue weight, mg
l—length of the optical path of the light beam, cm
ε—molar extinction coefficient, 5.6 µmol^−1^ × cm.

### 4.11. Determination of Endogenous Content of Free Proline

Extraction and determination of free proline were performed according to the method of Bates et al. [99] with minor modifications. A weighed sample of plant material weighing 0.1 g was triturated in liquid nitrogen. The ground material was transferred into test tubes and filled with 4 mL of distilled water. Test tubes with weighed samples were brought to a boil three times, cooling each time. The resulting extract was filtered into measuring tubes. The resulting extract was adjusted to the desired volume (6–7 mL) and then used for analysis. Tubes with 1 mL extract, 1 mL glacial acetic acid, 1 mL ninhydrin reagent (1.25 g ninhydrin, 20 mL 6 M H_3_PO_4_, 30 mL CH_3_COOH) were incubated for 1 h in a boiling water bath. Instead of the extract, 1 mL of distilled water was added to the control sample. The optical density of the obtained colored solutions was measured on a PD-303 spectrophotometer (Apel, Saitama, Japan) against the control at a wavelength of 520 nm. The content of proline was calculated using the calibration curve according to the formula:

C = Ek × V/(W × 1000),

where

C—proline concentration, µmol g^−1^ wet weight
E—optical density
k—coefficient calculated from the calibration curve
V—extract volume, mL
W—sample weight, g.

### 4.12. Determination of the Content of Soluble Sugars

To determine the total content of soluble sugars, frozen with liquid nitrogen and crushed plant leaves (150 mg sample) were subjected to extraction with 96% ethanol [100]. The homogenate was centrifuged (13,000× *g*, 10 min) and the total sugar content in the supernatant was determined spectrophotometrically by reaction with phenol and sulfuric acid (absorption at 490 nm). The calibration curve was built on sucrose.

To determine individual sugars, tissue fixation and extraction of sugars from plant tissue were performed with 80% ethanol. Quantitative determination of fructose and sucrose was carried out with resorcinol according to Roe [101]. The method is based on the formation of a colored compound during the interaction of ketose with resorcinol in an acidic medium, the color intensity of which was determined spectrophotometrically at a wavelength of 520 nm on a Pd-303 spectrophotometer (Apel, Kawaguchi, Japan) and the amount of fructose was calculated from calibration curves. Glucose was determined by the glucose oxidase method using a ready-made reagent kit (St. Louis, MO, USA) according to the producer’s instructions. The color intensity was determined at a wavelength of 530 nm using the same device. The sugar content was expressed in µmol per 1 g wet weight.

### 4.13. Determination of the Content of Phenolic Compounds and Flavonoids

Phenolic compounds were extracted with 96% ethanol from plant leaves frozen in liquid nitrogen and crushed (150 mg sample) for 40 min at 45 °C [102]. The homogenate was centrifuged (13,000× *g*, 10 min) and the content of the total phenolic compounds in the supernatant was determined by the spectrophotometric method with the Folin–Ciocolteu reagent at 725 nm [103] and the content of flavonoids with aluminum chloride at 415 nm [102]. The content of phenolic compounds in flavonoids was expressed in mg-Equiv. rutin/g fresh weight.

### 4.14. Determination of Phenolic Acids

To determine the content of phenolic (phenolcarboxylic) acids (chlorogenic, caffeic, ferulic) in the leaves of the tobacco different lines, 0.1 g of leaf tissue was homogenized with liquid nitrogen, 30 mL of 80% ethanol was added to the homogenate, and it was extracted for 1 h on a magnetic stirrer at 3–5 °C. After centrifugation at 10,000× *g* for 10 min, alcohol was again added to the sediment and the procedure was repeated two more times. The combined extract was filtered through a paper filter and evaporated under vacuum at 40 °C. The dry residue was dissolved in 2 mL of 96% ethanol. The obtained extract was purified by thin-layer chromatography on fluorescent silufol platinum CV 254 in an upward flow of solvent BUV: butyl alcohol: acetic acid: water (6:1:12). The zone of phenolic acids was determined by an external standard (Rf 0.9–0.8), collected, and eluted with 2 mL of 96% ethanol at 5 °C. The eluate was centrifuged; the supernatant was evaporated to dryness. To identify and quantify phenolic acids, the HPLC method was used on an isocratic column of Styer (Akvilon, Podolsk, Russia), an octodecyl RP-18 reverse phase column (Sigma, St. Louis, MO, USA), a mobile phase of acetonitrile: water: acetic acid (50:50:1), identification by an external standard, wavelength 254 nm. Statistical processing of results in Excel 2010 and Past V 3.0. Fivefold analytical repetition. The mean value of the indicators (M), the standard error of the mean (±SEM) and the confidence interval at the 95% confidence level were determined at *p* ≤ 5%.

### 4.15. Determination of the Content of Abscisic Acid

To determine the content of abscisic acid (ABA) 0.5 g of fresh leaves were fixed with liquid nitrogen, homogenized, and the homogenate was poured into 50 mL of boiling 96% ethanol. After cooling, the extract was poured off. The procedure was repeated, but already at 5 °C. The combined extract was filtered through a paper filter and evaporated under vacuum at 40 °C to an aqueous phase. The aqueous residue was basified to pH = 8.5, and purified with hexanol. The hexane fraction was discarded, the pH was adjusted to 3, and ABA was converted into the hexane fraction, which was evaporated to dryness, the dry residue was dissolved in 1 mL of acetone. Further purification was carried out by thin-layer chromatography on Silufol plates in an upward flow of CEA solvent: chloroform: ethyl acetate: acetic acid (100:100:1). The ABA RL zone was determined using an external standard. The spot was scraped off the plate and eluted in 2 mL of acetone for 3 h at 3 °C. The eluate was mixed, and centrifuged; the supernatant was evaporated to dryness. Identification and quantitation were carried out by HPLC using an external standard: on the isocratic system of instruments Styer (Akvilon, Podolsk, Russia), a column with reversed phase octadecyl RP-18, mobile phase acetonitrile: water, acetic acid (50:50:1).

### 4.16. Plant RNA Extraction

Total plant RNA was isolated by the trizol method by homogenizing the plant tissue with liquid nitrogen. RNA was purified from DNA impurities by treatment with DNase. The RNA concentration was measured on a Specol-11 spectrophotometer (Carl Zeiss, Jena, Germany) at a wavelength of 260 and 280 nm. The characteristics of the RNA integrity were assessed by melt curve analysis. The data shown in Figure S10. 

### 4.17. Gene Expression Analysis

PCR was performed in the final volume of 25 μL per sample using the 5× KTN-mix Polymerase (Evrogen, Moscow, Russia). DNA in the reaction mixture was amplified using the Thermal Cycler CFX96 Touch real-time amplifier (BioRad, Hercules, CA, USA). Amplification was performed in the following conditions: primary denaturation at 95 °C for 3 min; followed by 35 cycles of initial denaturation at 95 °C for 30 s, annealing at 64 °C for 30 s, and extension at 72 °C for 30 s.

The primers listed in Table S1 were used to analyze the *codA*, *Actin 2 and 9*, *P5CSA*, *ERD10B*, and *Zn/CuSOD* genes. 

### 4.18. Phenotyping Analisis

To identify phenotypic differences between genotypes, a Sinergotron ISR-02 phenoscanner (Institute for Development Strategy, Moscow, Russia) was used. Five potted plants of each variant were used for analysis. At least five measurements were taken from each sample. The analysis was performed using the following criteria: petiole shedding angle (leaves in the central region of the plant sample); color surface characteristics of the adaxial side (3–4 fully unfolded leaf); the presence of flowers, buds, as well as the absence of generative organs (Figure S6).

### 4.19. Transmission Electron Microscopy

For ultrastructural analysis, plant leaf segments about 1–2 mm in length and 0.5–1 mm in width were separated. Samples were fixed in a 2.5% glutaraldehyde solution in 0.1 M Sorensen phosphate buffer (pH 7.2) with the addition of 1.5% sucrose. After washing from the fixing mixture, the samples were post-fixed with a 1.0% solution of osmium tetroxide (OsO4), dehydrated in ethanol with increasing concentration (30, 50, 70, 96, and 100%), propylene oxide and encapsulated with Epon–Araldit epoxy resins. Semi-thin and ultrathin sections were prepared using the LKB–III ultramicrotome. Sections were contrasted with a 1% aqueous solution of uranyl acetate and lead citrate accordingly Reinolds, and analyzed at ×15,000 magnification using an H-500 transmission electron microscope (Hitachi, Tokyo, Japan). Images were processed in Adobe Photoshop 7.0.

### 4.20. Statistic Analysis

The results of physiological and biochemical studies carried out in at least five replicates were processed using Duncan’s tests with the corresponding errors (*p* = 0.05).

The compliance of the normality of the distribution was performed by the Kolmogorov–Smirnov method. The correlation coefficients were calculated using the Spearman method since not all analysis results had a normal distribution. Validation (significance) of the correlation coefficients was carried out by comparison with the standard values of the Student’s *t*-test at *p* = 0.01 and *p* = 0.05 significance levels.

## 5. Conclusions

Constitutive biosynthesis of the osmotically active compound glycine betaine led to a decrease in the content of proline and an indirect change in the synthesis of primary and secondary metabolites. We noted a slowdown in the formation of photosynthetic structures in leaf tissues and the transformation of prolamellar bodies into lamellar formations necessary for an intense photosynthetic process and responsible for the photosystem’s functioning. Phenotypically, this manifested itself in a weak intensity of green color in *codA* transgenic plants. Another important effect was the delay in the transition to generative development.

The predicted increase in salt tolerance of the processes associated with the most important biochemical molecular regulatory systems was confirmed similarly to many other studies conducted both using different methods of endogenous and exogenous delivery of glycine betaine. An important effect was the correction of the development rate in *codA* transgenic lines under salt exposure, the temp recovery of the structural organization of plastids, accompanied by an increase in the number of chlorophylls and regulation of the accompanying systems of primary and secondary metabolism. It can be assumed that the presence of an osmotically active compound not typical for tobacco not only affected the activity of enzymes and biosynthesis but also corrected the expression of genes associated with the response to salt stress. Probably this was indirectly the reason for a wider range of specific genomic modifications at the expression regulation level, which would be interesting to investigate in more specialized experiments in the future.

## Data Availability

Not applicable.

## Supplementary Materials

The supporting information can be downloaded at: https://www.mdpi.com/article/10.3390/ijms241813998/s1.
