# Supplementary material for: Heterologous codA Gene Expression Leads to Mitigation of Salt Stress Effects and Modulates Developmental Processes
Source: Int J Mol Sci. 2023 Sep 12;24(18):13998. doi: 10.3390/ijms241813998 (PMC10531037; doi:10.3390/ijms241813998)
Supplement: Supplementary file 1 [file ijms-24-13998-s001.zip › ijms-2551232-supplementary.pdf]

Heterologous *codA* Gene Expression Leads to Mitigation of Salt Stress Effects and Modulates Developmental Processes

Supplementary Materials

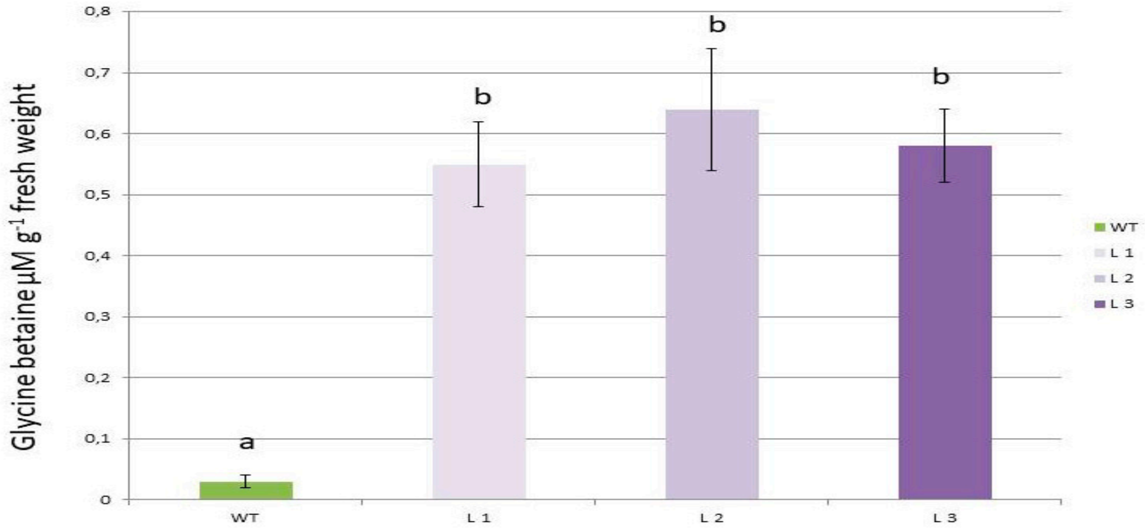

Supplementary Materials Figure S1. Glycine betaine content in transgenic plant lines. Control—WT tobacco plants; L1, L2, and L3—codA transgenic lines. Columns with the same letters are not significantly different ($p \leq 0.05$, Duncan's test).

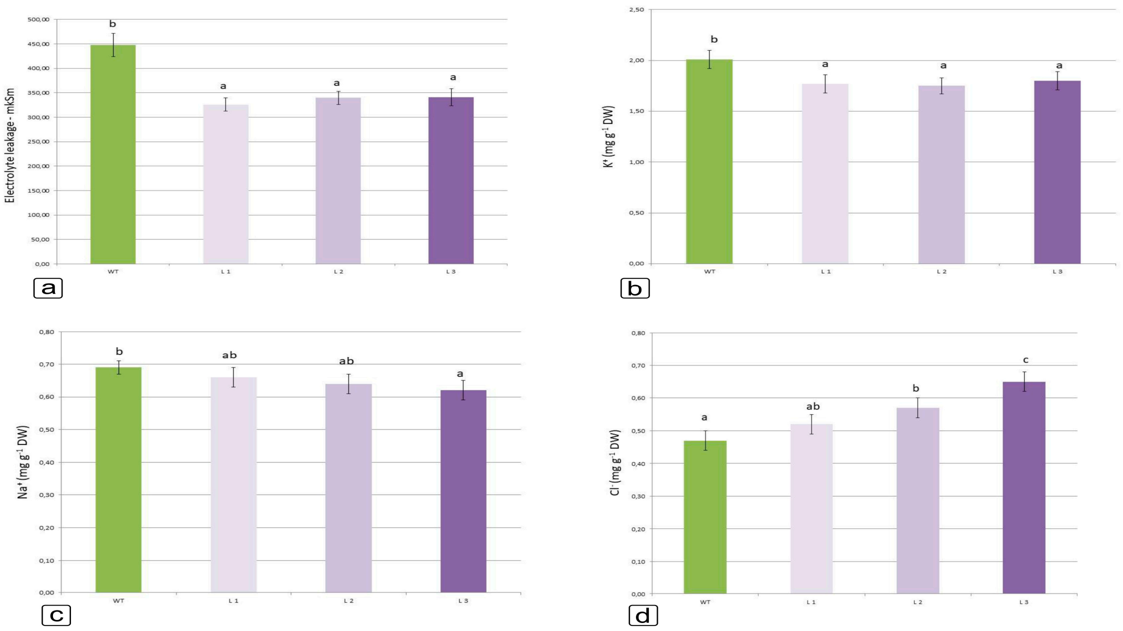

Supplementary Materials Figure S2. Characterization of the physiological and biochemical features of electrolyte outflow (a), the content of K (b), Na (c), and Cl (d) ions in wild-type plants and *codA* transgenic tobacco plants with constitutive expression of choline oxidase gene.

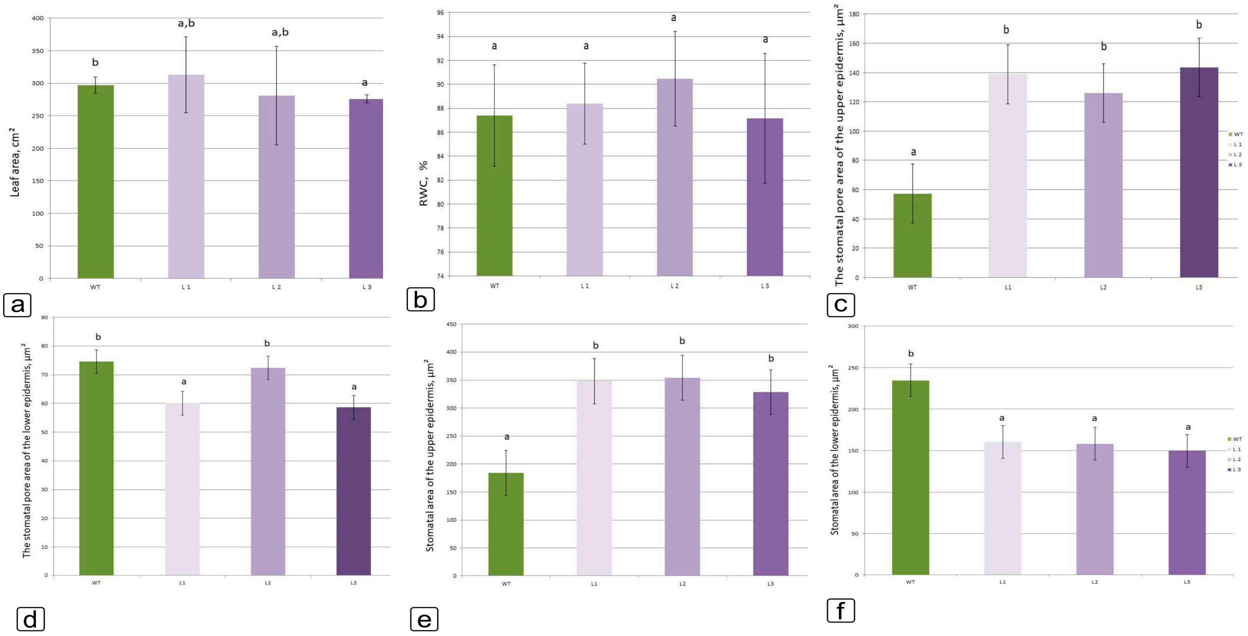

Supplementary Materials Figure S3. Characterization of the physiological and biochemical features of leaf area (a), hydration (b), size of stomata pore (c,d) and stomata cells (e,f) of upper (c,e) and lower(d,f) epidermis in wild-type plants and *codA* transgenic tobacco lines with constitutive expression of choline oxidase gene.

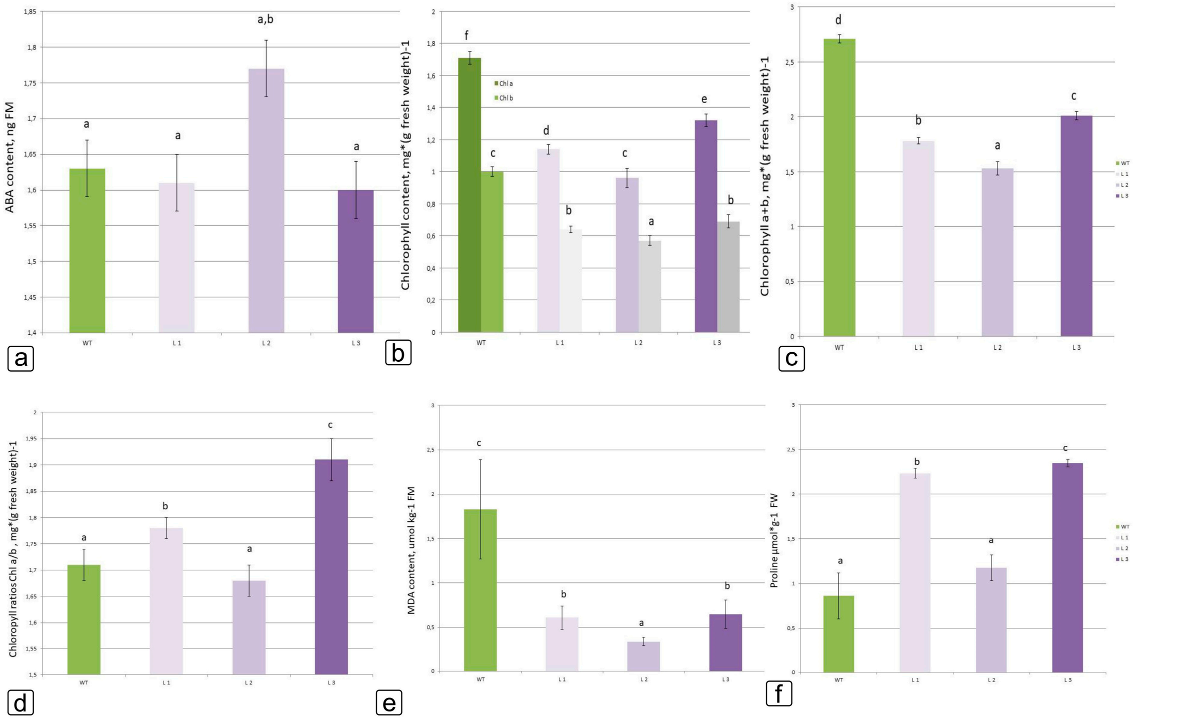

Supplementary Materials Figure S4. Characterization of the physiological and biochemical features of ABA (a), chlorophylls a and b (b), a+b sum (c) and their ratios (d), as well as MDA (e) and proline (f) in wild-type plants and *codA* transgenic tobacco lines with constitutive expression of choline oxidase gene.

Supplementary Materials Figure S5. Characterization of the physiological and biochemical

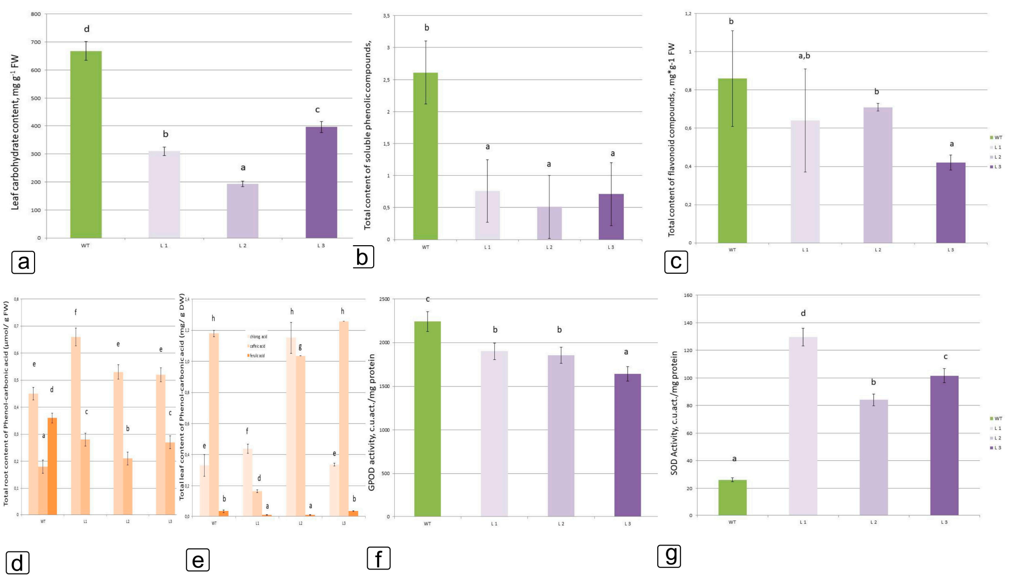

features of free sugar (a), phenolic compounds (b), flavonoids (c) and phenolic acids (chloric, caffeic and ferulic) in root (d) and leaf (e), as well as gPOD (f) and SOD (g) activity in wild-type plants and *codA* transgenic tobacco lines with constitutive expression of choline oxidase gene.

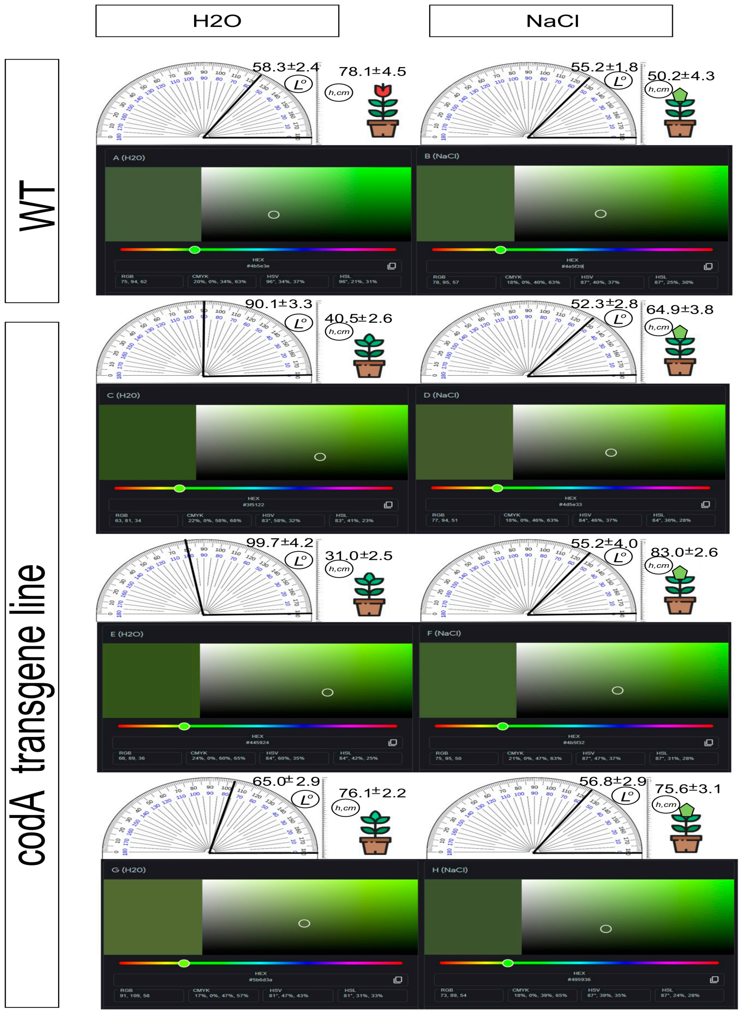

Supplementary Materials Figure S6. Results of phenotyping of control and *codA* transgenic lines of tobacco plants with assessment of plant height, angle of deviation of leaf petioles in the central part of plants, transition of plants to budding and flowering, green mass color parameters. Averaged data are given based on the results of measuring 5

independent plants of each variant during cultivation using water and NaCl solution (up to a final concentration of 200 mM NaCl based on the total moisture capacity of the substrate).

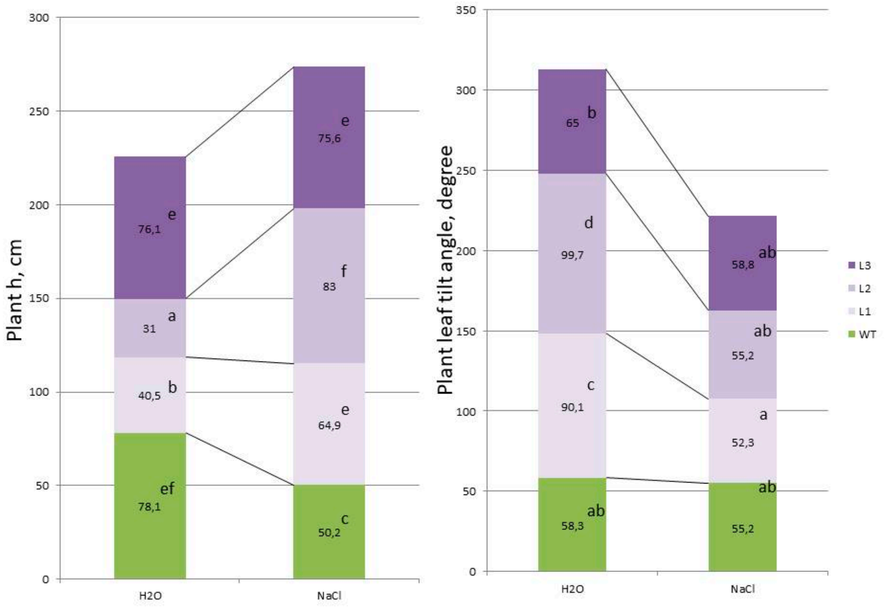

Figure S7. Characterization of the plant height and degree of deviation of leaf petioles from the stem in response to 200 mM NaCl salinity in WT plants and *codA* transgenic tobacco plants.

Gene	Treatment	Line	Leaf area sm2	RWC	Area of the stomatal fissure of the upper epidermis, μm2	Area of the stomatal fissure of the lower epidermis, μm2	Area of stomata of the lower epidermis, μm2	GB content	ABA content, ng FM	Chloropyll a, mg*(g fresh weight)-1	Chloropyll b, mg*(g fresh weight)-1	Chloropyll a+b, mg*(g fresh weight)-1	Chloropyll a/b, mg*(g fresh weight)-1	Elecrtolite leakage-mkSm	K+	Na2+
NtAct9PBL	Control (H2O)	WT	0,500	0,000	0,100	-0,300	1,000**	0,224	0,600	-0,667	0,051	0,667	-0,100	-0,400	0,100	0,359
		L1	0,462	0,103	0,872	0,821	-0,564	-,895*	0,289	0,205	-0,667	-0,359	0,205	0,667	0,205	-0,289
		L2	0,667	0,667	0,821	0,667	-0,359	-0,237	0,658	-0,289	0,205	-0,103	0,051	-0,872	-0,718	-0,289
		L3	-0,395	-0,051	0,053	0,051	0,359	0,711	-0,500	0,308	-0,667	-0,410	0,359	0,667	1,000**	0,053
	NaCl	WT	-,921*	,975**	0,051	-0,872	-0,410	0,649	-0,564	0,103	,947*	-0,462	-0,667	0,564	-0,616	0,154
		L1	0,500	0,800	-0,300	-0,154	0,000	1,000**	0,000	-,900*	-0,718	-0,600	-0,821	-0,600	-0,200	0,600
		L2	-0,700	0,300	-0,700	0,000	-1,000**	0,667	0,667	-0,051	-0,300	0,400	,975**	0,100	-0,200	-0,462
		L3	0,462	0,359	0,263	0,410	0,763	-0,564	-0,132	0,395	-0,359	-0,205	0,289	-0,103	-0,359	-0,821
NtP5CS A	Control (H2O)	WT	-0,700	0,300	0,800	-0,400	-0,400	-,894*	0,200	-0,103	-0,667	-0,667	0,700	1,000**	-0,700	0,564
		L1	0,300	0,500	0,200	0,500	-0,600	-0,051	0,872	-0,400	0,000	0,600	0,800	0,300	,900*	0,564
		L2	-0,600	-0,600	-0,200	0,300	-0,100	0,872	0,205	0,718	0,000	,900*	1,000**	-0,500	-0,100	0,410
		L3	0,410	-0,200	0,359	0,500	-0,100	-0,667	-0,205	0,700	0,300	0,500	-0,100	-0,300	-0,359	0,359
	NaCl	WT	,921*	-,975**	-0,103	,975**	0,667	-0,433	0,359	0,051	-,895*	0,359	0,821	-0,410	0,821	-0,051
		L1	0,700	0,600	0,100	0,205	0,100	,900*	-0,400	-0,800	-0,872	-0,700	-,975**	-0,800	0,100	0,700
		L2	-0,100	-0,600	-0,100	-0,600	-0,100	0,205	0,667	-0,051	,900*	-0,200	0,205	-0,200	0,100	-0,462
		L3	0,154	0,205	0,026	0,103	,921*	-0,410	-0,368	0,553	-0,205	-0,359	0,053	-0,718	-0,667	-0,667
NtDHN	Control (H2O)	WT	-0,600	-0,100	,900*	-0,300	0,300	-0,783	0,500	-0,821	-0,872	-0,410	,900*	0,600	-,900*	,975**
		L1	0,359	0,103	0,564	0,308	-0,154	-0,579	-0,237	-0,103	-0,667	-0,564	-0,103	-0,051	-0,410	-0,395
		L2	-0,400	-0,400	-0,300	-0,300	0,100	0,154	-0,205	0,821	-0,500	0,600	0,500	-0,500	0,100	0,103
		L3	кспрессии													
	NaCl	WT	0,289	-0,410	0,359	0,564	0,308	-0,649	0,821	-0,821	-0,763	,975**	0,410	0,154	0,462	-0,462
		L1	-0,051	-0,154	-0,359	-,921*	0,616	-0,103	0,359	-0,051	0,684	0,821	0,368	0,667	-0,667	-0,821
		L2	-0,359	0,359	0,103	0,564	0,308	0,289	0,026	-,921*	0,154	0,051	-0,395	0,051	-0,821	-0,289
		L3	-,975**	-0,205	-0,553	-0,718	0,026	0,821	0,368	-0,026	0,205	-0,667	0,158	-0,308	0,051	0,051
SODLC1	Control (H2O)	WT	-0,500	0,500	,900*	-0,700	0,000	-0,783	0,400	-0,359	-0,564	-0,410	0,600	,900*	-0,600	0,667
		L1	-,900*	-0,800	-0,500	-,900*	1,000**	0,359	-0,564	0,300	0,200	0,200	-,900*	-0,500	-0,500	0,154
		L2	-0,700	-0,700	-,900*	-0,600	-0,200	-0,154	-,975**	0,359	0,100	0,300	-0,100	0,700	1,000**	-0,359
		L3	-,975**	-0,400	0,205	0,000	-0,700	0,718	0,359	-0,600	0,100	-0,500	-0,700	-0,100	0,308	0,205
	NaCl	WT	0,237	-0,410	0,564	0,667	0,564	-0,351	0,564	-0,667	-0,763	0,821	0,667	0,359	0,564	-0,103
		L1	0,154	0,821	-0,667	-0,553	0,205	0,821	0,205	-0,667	-0,368	-0,103	-0,526	-0,308	-0,718	0,103
		L2	0,564	-0,616	0,410	-0,872	0,154	-0,395	0,000	,947*	0,359	-0,564	0,026	0,103	,975**	0,158
		L3	-0,821	-0,667	-0,789	-0,872	-0,289	,975**	0,132	0,132	0,667	-0,205	-0,237	0,154	0,667	0,205
ChSYNPBL	Control (H2O)	WT	0,527	-0,264	-0,580	0,264	0,527	0,825	-0,211	-0,189	0,541	0,541	-0,527	-,949*	0,527	-0,379
		L1	0,600	0,300	,900*	,900*	-0,700	-0,872	0,410	0,000	-0,700	-0,300	0,400	0,600	0,300	-0,205
		L2	0,616	0,616	0,359	0,205	-0,410	-0,763	-0,053	-,895*	0,564	-0,718	-0,821	0,359	0,051	-0,684
		L3	-0,410	0,200	-0,359	-0,500	0,100	0,667	0,205	-0,700	-0,300	-0,500	0,100	0,300	0,359	-0,359
	NaCl	WT	0,821	-,900*	0,000	1,000**	0,800	-0,316	0,300	0,000	-0,872	0,400	,900*	-0,200	,900*	0,000
		L1	-0,300	-0,400	0,500	0,872	-0,400	-0,600	-0,400	0,700	-0,103	-0,200	0,205	-0,200	0,600	0,200
		L2	-,900*	0,400	-0,600	0,100	-,900*	0,872	0,821	-0,410	-0,100	0,300	0,872	0,200	-0,500	-0,718
		L3	0,154	-0,821	-0,500	-0,410	-0,132	0,103	-,895*	0,553	0,821	0,667	-1,000**	-0,205	0,359	0,359
codA	Control (H2O)	WT	не было экспрессии гена													
		L1	-0,224	0,112	0,112	-0,224	0,224	0,000	0,344	-0,783	-0,447	0,671	0,112	-0,671	0,224	0,803
		L2	0,400	0,400	0,300	-0,300	,900*	0,051	0,410	-0,205	-0,700	-0,600	-0,300	0,100	-0,500	0,718
		L3	-0,342	0,718	-0,789	-0,821	-0,154	-0,263	0,763	-0,205	-0,564	0,667	-0,154	-0,667	0,000	-0,789
	NaCl	WT	не было экспрессии гена													
		L1	-0,600	0,300	-0,200	0,564	-0,300	-0,200	-0,300	0,600	-0,359	-0,100	-0,051	-0,500	-0,200	0,100
		L2	0,700	-0,300	0,700	0,000	1,000**	-0,667	-0,667	0,051	0,300	-0,400	-,975**	-0,100	0,200	0,462
		L3	0,051	0,564	0,368	0,308	0,553	-0,462	-0,026	-0,026	-0,564	-0,410	0,289	-0,821	-,975**	-0,205

Gene	Treatment	Line	Cl-	Contents of MDA	Proline content	Carbohydrate content	Total content of soluble phenolic compounds	flavonoids	roots.chlorog. acid	roots.caffeic acid	roots.ferulic acid	leaf.chlorog. acid	leaf.caffeic acid	leaf.ferulic acid	POD activity	SOD activity
NtAct9PBL	Control (H2O)	WT	-0,200	-0,051	0,400	-0,400	0,400	-0,300	0,000	-0,300	-0,300	-0,100	-0,800	-0,600	-,900*	-0,100
		L1	0,872	-0,132	-0,359	-,975**	-0,816	0,154	-0,462	-0,872		0,154	-0,103	0,410	-0,718	-0,872
		L2	-0,051	0,763	-0,718	-0,359	-0,051	0,462	0,763	-0,205		0,821		0,763	-0,462	-0,103
		L3	-0,564	0,821	0,872	-0,564	-0,667	-0,205	0,564	0,051		-,921*		-0,649	-,975**	-0,821
	NaCl	WT	0,564	0,154	-0,205	,975**	-,975**	0,553	-0,263	0,462	-0,462	0,667	-0,051	-0,667	0,051	0,564
		L1	0,300	-0,700	-0,100	-1,000**	-0,718	1,000**	0,051	0,000		0,308	0,205	-1,000**	-0,100	-0,400
		L2		0,500	-0,300	0,500	-0,400	0,462	-0,700	-0,103		0,200		-0,667	-1,000**	-0,200
		L3	0,410	0,205	0,154	-0,205	-0,051	0,368	-,921*	0,205		,975**		0,289	0,205	,975**
NtP5CS A	Control (H2O)	WT	0,600	0,667	0,500	-0,300	0,500	,900*	,900*	,900*	,900*	0,700	0,100	0,700	0,200	0,300
		L1	-0,100	-0,154	-0,800	-0,100	0,051	0,200	0,600	0,100		-0,300	-,900*	-,900*	-0,600	-0,200
		L2	-1,000**	0,205	-0,100	0,500	-1,000**	,900*	0,205	0,300		-0,200		-0,103	-,900*	-0,300
		L3	0,600	-0,100	0,100	0,600	0,300	0,300	-0,600	0,200		0,616		0,000	0,500	0,100
	NaCl	WT	-0,359	-0,051	-0,051	-0,872	0,872	-0,763	0,368	-0,667	0,667	-0,821	-0,205	0,462	-0,359	-0,359
		L1	0,600	-0,600	0,300	-,900*	-0,872	,900*	0,051	-0,400		-0,051	-0,154	-,900*	-0,300	-0,200
		L2		0,200	0,600	-0,100	0,200	0,462	-0,100	,975**		-0,100		0,410	-0,100	0,100
		L3	0,103	0,359	0,462	-0,667	-0,205	-0,026	-0,684	0,359		0,821		0,684	0,359	0,667
NtDHN	Control (H2O)	WT	0,000	0,872	,900*	-0,500	,900*	0,800	0,700	0,800	0,800	,900*	-0,700	-0,100	-0,600	0,500
		L1	0,564	-0,026	0,051	-0,667	-0,395	-0,359	-0,359	-0,564		0,051	0,205	0,821	-0,205	-0,564
		L2	-0,500	0,308	0,100	0,000	-0,500	0,600	0,308	0,200		-0,300		0,359	-0,600	-0,200
		L3														
	NaCl	WT	-0,821	-0,462	0,667	-0,667	0,667	-0,342	-0,395	-0,205	0,205	-0,410	0,410	0,410	0,103	-0,821
		L1	-0,051	0,564	-0,205	0,103	0,684	-0,103	0,763	0,359		0,684	0,368	0,103	-0,359	0,462
		L2		0,667	-0,359	0,051	-0,051	0,289	-0,359	0,158		-0,205		0,289	0,308	-0,821
		L3	-0,718	0,051	-0,462	-0,564	0,616	-0,816	0,684	0,667		-0,410		0,579	0,667	-0,667
SODLC1	Control (H2O)	WT	0,700	0,564	0,600	-0,600	0,600	0,800	1,000**	0,800	0,800	0,600	-0,200	0,600	-0,100	0,100
		L1	-0,200	0,667	0,600	0,700	0,154	-0,400	0,000	0,200		0,600	0,700	0,200	0,700	0,500
		L2	0,100	-0,154	1,000**	-0,300	0,100	-0,300	-0,154	-0,400		-,900*		-0,103	0,300	-0,500
		L3	-0,800	-0,200	0,200	-0,800	0,100	0,600	0,800	0,400		-0,564		0,264	-0,500	0,200
	NaCl	WT	-0,564	-0,103	0,410	-0,616	0,616	-0,395	-0,079	-0,205	0,205	-0,667	0,051	0,103	-0,205	-0,564
		L1	0,051	-0,359	-0,359	-0,821	-0,368	0,821	0,342	0,205		0,789	0,632	-0,821	-0,051	-0,205
		L2		-0,821	0,616	-0,103	0,564	-0,158	0,564	0,263		-0,205		0,026	0,154	,975**
		L3	-0,872	-0,564	-0,616	0,051	0,462	-0,816	,921*	0,205		-0,564		0,026	0,205	-0,821
ChSYNPBL	Control (H2O)	WT	-0,632	-0,541	-0,369	0,053	-0,369	-0,738	-0,791	-0,738	-0,738	-0,527	-0,369	-0,791	-0,369	-0,316
		L1	0,800	-0,205	-0,500	-1,000**	-0,718	0,100	-0,300	-0,800		0,000	-0,300	0,300	-0,800	-,900*
		L2	0,821	0,158	0,051	-0,667	0,821	-0,667	0,158	-0,667		0,359		0,289	0,667	-0,103
		L3	-0,600	0,100	-0,100	-0,600	-0,300	-0,300	0,600	-0,200		-0,616		0,000	-0,500	-0,100
	NaCl	WT	-0,300	0,000	-0,100	-0,800	0,800	-0,821	0,359	-0,700	0,700	-,900*	-0,300	0,300	-0,500	-0,300
		L1	-0,100	0,100	0,300	0,600	-0,103	-0,600	-0,564	-0,400		-0,718	-0,410	0,600	0,300	0,000
		L2		0,800	-0,400	0,600	-0,300	0,718	-,900*	0,103		0,000		-0,564	-,900*	-0,500
		L3	-0,410	-0,667	0,462	0,359	-0,718	-0,289	0,368	-0,667		-0,205		-0,368	-0,667	-0,359
codA	Control (H2O)	WT														
		L1	-0,112	0,574	-0,447	0,224	0,229	-,894*	,894*	0,112		0,112	-0,447	-0,335	-0,112	-0,112
		L2	0,300	-0,718	-0,500	0,600	0,300	-0,400	-0,718	0,800		0,300		-0,564	0,400	1,000**
		L3	0,308	0,205	0,103	0,308	0,667	-0,205	-0,308	-0,718		0,079		0,676	-0,051	0,410
	NaCl	WT														
		L1	-0,300	0,000	-0,100	0,200	-0,359	-0,200	-0,359	-0,300		0,051	0,359	0,200	0,500	-0,100
		L2		-0,500	0,300	-0,500	0,400	-0,462	0,700	0,103		-0,200		0,667	1,000**	0,200
		L3	0,308	0,821	0,564	-0,821	-0,154	0,105	-0,500	0,410		0,359		0,711	0,410	0,359

Gene	Treatment	Line	expression level NtAct9PBL	expression level NtP5CS A	expression level NtDHN	expression level SODLC1	expression level ChSYNPBL	expression level codA	PlantHcm	L	GeneCodA	R	G	B	S
NtAct9PBL	Control (H2O)	WT	1,000	-0,400	0,300	0,000	0,527		-0,300	0,600	0,500	-0,300	,894*	0,154	0,718
		L1	1,000	-0,051	0,684	-0,564	,975**	-0,344	-0,553	0,359	-1,000**	0,667	0,667	0,667	0,359
		L2	1,000	0,051	0,205	-0,718	0,132	-0,103	0,462	-0,051	1,000**	-0,564	-0,564	-0,658	-0,026
		L3	1,000	-0,359		0,308	0,359	0,000	,975**	-0,667	1,000**	0,684	,975**	-0,154	0,289
	NaCl	WT	1,000	-,947*	-0,553	-0,526	-0,872			-,975**	-0,866	-0,344	0,363	-0,667	0,632
		L1	1,000	,900*	-0,103	0,821	-0,600	-0,200		0,200	0,500	-0,872	-0,718	-0,872	,949*
		L2	1,000	0,100	-0,308	-0,154	,900*	-1,000**	0,300	0,300	-0,500	-0,462	-0,359	-0,359	-0,410
		L3	1,000	0,763	-0,553	-0,711	-0,289	0,368	-0,821	,975**	-1,000**	0,410	0,500	0,410	-0,205
NtP5CS A	Control (H2O)	WT	-0,400	1,000	0,600	,900*	-,949*		0,100	0,000	-1,000**	-0,500	-0,224	0,359	-0,359
		L1	-0,051	1,000	-0,564	-0,600	0,100	0,224	0,051	-0,600	-0,500	-0,400	-0,400	-0,700	0,800
		L2	0,051	1,000	0,500	-0,100	-0,821	-0,300	0,000	0,200	1,000**	-0,600	-0,600	-0,667	,975**
		L3	-0,359	1,000		-0,500	-1,000**	-0,154	-0,500	,900*	0,500	-0,462	-0,200	-0,200	0,154
	NaCl	WT	-,947*	1,000	0,500	0,553	,975**			,975**	1,000**	0,574	-0,181	0,821	-0,763
		L1	,900*	1,000	-0,359	0,564	-0,300	-0,100		0,100	0,500	-,975**	-0,821	-,975**	,949*
		L2	0,100	1,000	0,154	0,154	0,300	-0,100	0,600	0,600	0,500	-0,462	-0,667	0,103	-0,872
		L3	0,763	1,000	-0,158	-0,553	-0,053	0,763	-0,667	0,821	-0,500	0,564	0,500	0,564	-0,667
NtDHN	Control (H2O)	WT	0,300	0,600	1,000	0,700	-0,369		0,300	0,100	-0,500	-0,800	0,224	0,103	0,359
		L1	0,684	-0,564	1,000	-0,154	0,667	0,000	-0,079	0,564	0,500	,975**	,975**	,975**	-0,051
		L2	0,205	0,500	1,000	0,100	-0,718	-0,200	0,000	-0,700	0,500	0,100	0,100	-0,103	0,564
		L3													
	NaCl	WT	-0,553	0,500	1,000	,921*	0,564			0,410	-0,500	0,000	0,363	0,410	-0,263
		L1	-0,103	-0,359	1,000	0,368	-0,718	-0,462		0,718	-0,500	0,526	0,763	0,526	-0,162
		L2	-0,308	0,154	1,000	-0,789	0,103	0,308	-0,667	0,359	1,000**	-0,395	-0,526	-0,263	0,026
		L3	-0,553	-0,158	1,000	0,763	-0,158	0,079	0,667	-0,410	1,000**	-0,564	-0,711	-0,564	-0,564
SODLC1	Control (H2O)	WT	0,000	,900*	0,700	1,000	-0,791		-0,200	0,400	-0,500	-0,700	0,224	0,616	-0,205
		L1	-0,564	-0,600	-0,154	1,000	-0,700	0,224	-0,103	-0,200	1,000**	-0,300	-0,300	0,000	-0,600
		L2	-0,718	-0,100	0,100	1,000	0,051	-0,500	-,900*	-0,100	-0,500	0,700	0,700	0,667	0,103
		L3	0,308	-0,500		1,000	0,500	0,205	0,500	-0,700	0,500	0,103	0,100	0,100	-0,205
	NaCl	WT	-0,526	0,553	,921*	1,000	0,667			0,410	-0,500	0,344	0,363	0,667	-0,553
		L1	0,821	0,564	0,368	1,000	-0,821	-0,103		0,616	0,500	-0,500	-0,263	-0,500	0,649
		L2	-0,154	0,154	-0,789	1,000	-0,410	0,154	0,667	-0,103	-1,000**	0,632	0,579	0,711	-0,237
		L3	-0,711	-0,553	0,763	1,000	0,237	-0,553	,975**	-0,564	1,000**	-0,872	-,947*	-0,872	0,051
ChSYNPBL	Control (H2O)	WT	0,527	-,949*	-0,369	-0,791	1,000		-0,105	0,105	1,000**	0,211	0,354	-0,270	0,406
		L1	,975**	0,100	0,667	-0,700	1,000	-0,224	-0,410	0,300	-1,000**	0,700	0,700	0,600	0,500
		L2	0,132	-0,821	-0,718	0,051	1,000	-0,103	-0,205	0,308	-1,000**	0,205	0,205	0,289	-0,789
		L3	0,359	-1,000**		0,500	1,000	0,154	0,500	-,900*	-0,500	0,462	0,200	0,200	-0,154
	NaCl	WT	-0,872	,975**	0,564	0,667	1,000			,900*	1,000**	0,671	0,000	,900*	-0,821
		L1	-0,600	-0,300	-0,718	-0,821	1,000	0,600		-0,600	0,500	0,154	-0,103	0,154	-0,527
		L2	,900*	0,300	0,103	-0,410	1,000	-,900*	0,100	0,600	0,500	-0,616	-0,616	-0,359	-0,564
		L3	-0,289	-0,053	-0,158	0,237	1,000	-0,289	0,359	-0,205	-0,500	0,051	-0,026	0,051	0,359
codA	Control (H2O)	WT													
		L1	-0,344	0,224	0,000	0,224	-0,224	1,000	0,287	-0,671	0,866	0,112	0,112	-0,112	0,447
		L2	-0,103	-0,300	-0,200	-0,500	-0,103	1,000	0,800	-0,300	0,500	0,100	0,100	0,205	-0,462
		L3	0,000	-0,154		0,205	0,154	1,000	0,051	-0,154	0,000	0,632	-0,051	,975**	-,947*
	NaCl	WT													
		L1	-0,200	-0,100	-0,462	-0,103	0,600	1,000		0,000	1,000**	-0,051	-0,154	-0,051	-0,369
		L2	-1,000**	-0,100	0,308	0,154	-,900*	1,000	-0,300	-0,300	0,500	0,462	0,359	0,359	0,410
		L3	0,368	0,763	0,079	-0,553	-0,289	1,000	-0,616	0,359	0,500	0,718	0,605	0,718	-0,821

Figure S8. Spearman's correlation coefficients between indicators of gene expression and the main morphometric and biochemical parameters. Legend: only significant correlation coefficients are highlighted, * - correlation is significant at p=0.05, ** - correlation is significant at p=0.01; dark blue - very strong inverse (negative) correlation (r<-0.9), blue -

strong inverse (negative) correlation (r<-0.8), dark orange - very strong direct (positive) correlation (r>0.9), orange - strong direct (positive) correlation (r>0.8).

Figure S9. The choline oxidase expression cassette. Обозначения: RB and LB—right and left border sequences of T-DNA; codA—choline oxidase gene with transit plastid targeted peptide sequence (pls); NPTII—neomycin phosphotransferase gene; NOS pro—nopaline synthase promoter; NOS ter—nopaline synthase terminator; 35S pro—promoter of cauliflower mosaic virus CaMV 35S RNA.

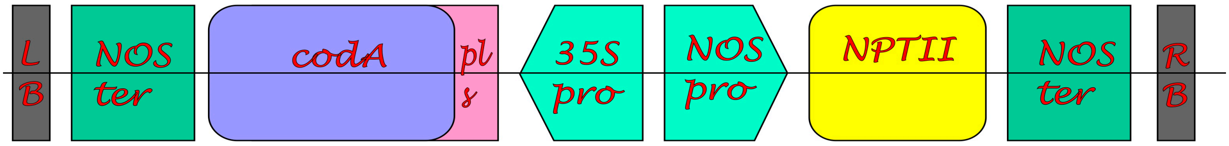

Table S1. The primers used in the gene expression analysis

	Title	Sequence 5` → 3`	Primer length	T°
codA	codA_F	TGCACCTGATGAACGTGCC	19	58-
	codA_R	GGTGGAGAGCACGACTTCAT	20	60

	Title	Sequence 5` → 3`	Primer length	T°
Actin	NtAct2_F	TGCACCTGATGAACGTGCC	19	58-
	NtAct2_R	GGTGGAGAGCACGACTTCAT	20	60
	NtAct9PBL_F	TCGCTGATCGAGTCCAATCTG	20	60
	NtAct9PBL_R	GCATGAACAACGACGTGCAA	20	

	Title	Sequence 5` → 3`	Primer length	T°
P5CS A	NtP5CS A_F	TGCACCTGATGAACGTGCC	19	60
	NtP5CS A _R	GGTGGAGAGCACGACTTCAT	20	

	Title	Sequence 5` → 3`	Primer length	T°
ERD10B	DehF2_F	ATGGACAAGGCGGAAGAAG	20	60
	DehR2_R	GTTGTTGCAGTTGAATGAGT	19	

	Title	Sequence 5` → 3`	Primer length	T°
Zn/CuSOD	SODLC1_F	CACAGTTACTGGAAATGTCTCT	22	60

	SODLC1_R	GATTGTAATGTGGTCCCGTTGA	22	

	Title	Sequence 5` → 3`	Primer length	T°
ChSyn	ChSYNPBL1F	TCTCTGCAGCCCAAACTCTT	20	58-60
	ChSYNPBL1R	GTTGGAATGCTTCCACAAGGC	22	

Figure S10.

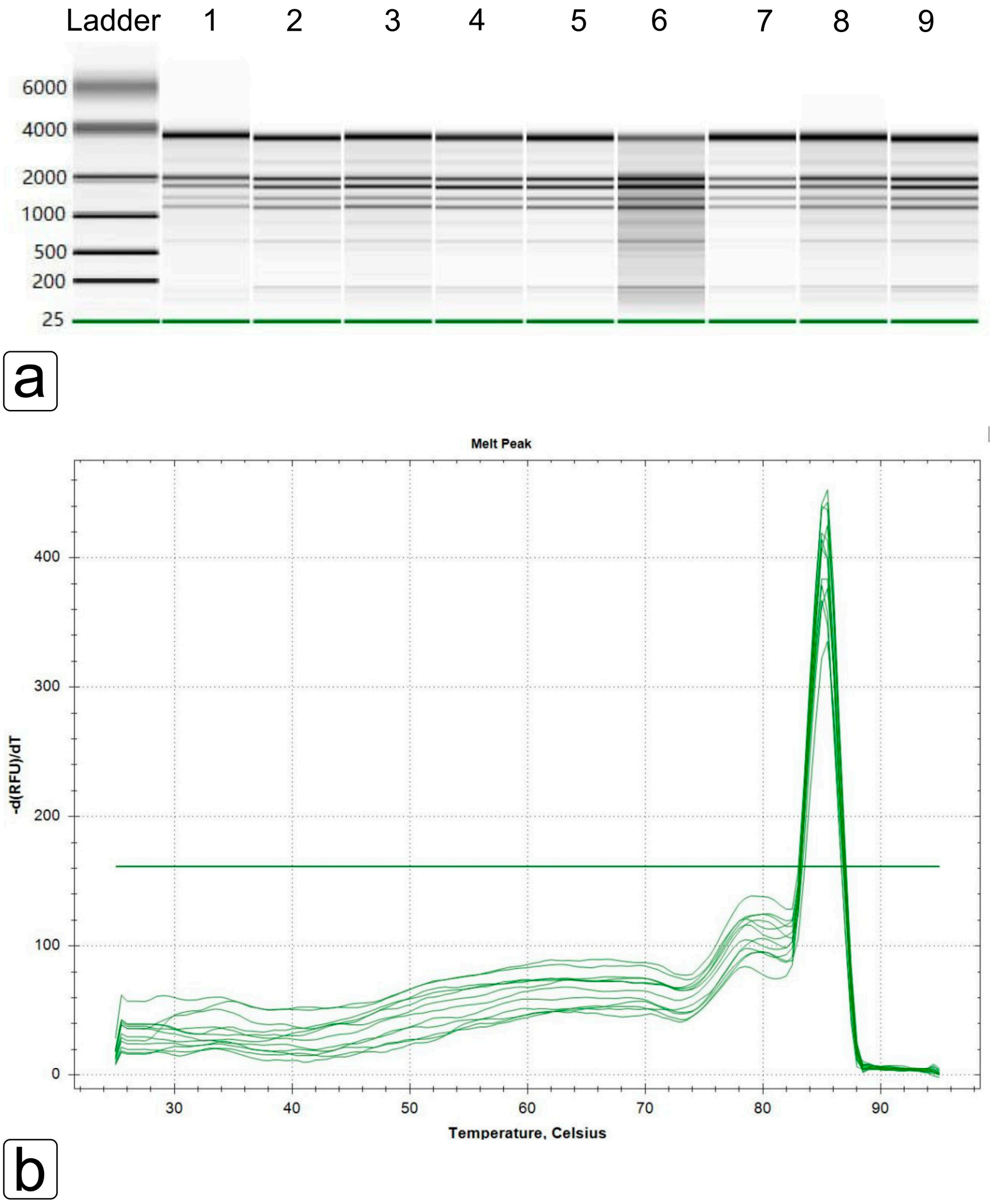

Figure S10. Integrity of RNA samples isolated from leaf tissue of tobacco plants.

(a) Electrophoregram of RNA ladder and total RNA isolated by trizol method.

(b) Melt curve analysis. The SYBR Green qPCR reactions contained 10 ng RNA-equivalent cDNA from tobacco leaf tissue. The reactions were performed with CFX96 Touch real-time amplifier. The reaction mixtures contained variable primer pair concentrations.
